# Manipulating D–A interaction to achieve stable photoinduced organic radicals in triphenylphosphine crystals†

**DOI:** 10.1039/d2sc05753k

**Published:** 2023-01-18

**Authors:** Chunlin Tang, Lijuan Song, Kang Zhou, Peng Ren, Engui Zhao, Zikai He

**Affiliations:** a School of Science, Harbin Institute of Technology Shenzhen Guangdong 518055 China hezikai@hit.edu.cn; b Hoffmann Institute of Advanced Materials, Shenzhen Polytechnic Shenzhen Guangdong 518055 China; c School of Chemical Engineering and Technology, Harbin Institute of Technology Harbin Heilongjiang 150001 China

## Abstract

New strategies for the design and synthesis of stable organic radicals without additives are highly desirable. Herein, we design a series of donor–acceptor structured triarylphosphines and disclose the fast color change triggered by UV-irradiation in the crystalline state. Photoinduced organic radicals are undoubtedly verified and proved to be the reason for the color change by time-dependent and quantitative electron paramagnetic resonance analysis, X-ray crystallographic analysis, and theoretical calculations. It is revealed that the intrinsic symmetry breaking of peripheral architecture helps to form continuous molecular chains by donor–acceptor counterpart pairing. Intermolecular electron-transfer occurs among molecular chains and results in radical ion pairs upon photoirradiation.

## Abstract 2

Herein, we realized the quick generation of stable photoinduced radicals in donor–acceptor triarylphosphines with associated color changes of emission and absorption, which was deciphered by the electron paramagnetic resonance (EPR) spectra.

## Graphical abstract

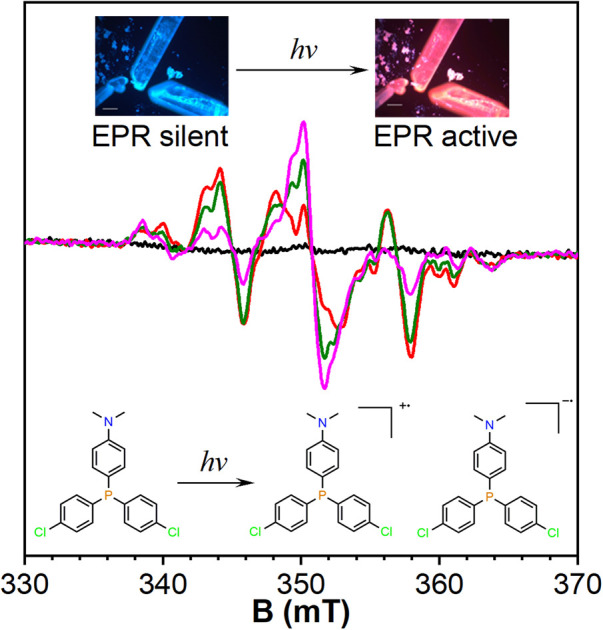

## Introduction

Photoinduced organic radicals are important for understanding the chemical and physical processes of functional materials. They have been widely investigated and found to have versatile applications in the fields of organic synthesis,^1–4^ photoelectronic devices,^5,6^ and biotechnology.^7–9^ Extensive attempts have been made to investigate the photophysical properties of these sensitive radicals. For example, there are many examples for nitrogen-containing photochromic systems, such as hexaarylbiimidazole derivatives,^10^ 2,4,6-tri(4-pyridyl)-1,3,5-triazine,^11^ phenothiazine-poly(dimethylsiloxane) hybrids,^12^ tri-*p*-tolylamine,^13–15^*etc.* However, most radical species are generated slowly over hours of continuous photoirradiation and are quite unstable under ambient conditions. As a result, the development of new stable photoinduced radicals is still challenging.

Triarylphosphines are widely used as ligands for transition metal catalyzed cross coupling^16–18^ and redox centers of functional materials.^19–22^ These electron-rich triarylphosphines can undergo facile oxidation to form radical cations.^23–26^ Pure triarylphosphine radical cations have been isolated and undoubtedly characterized.^27,28^ However, these radicals are often transient or unstable intermediates in the solid state because of their high reactivity. Although bulky carbene ligands^24,29,30^ and weakly coordinating anions^27,28^ were reported to partially stabilize them, stable triarylphosphine radicals are still hardly achieved.^31^ To date, there is only one literature study which reports that tris(4-chlorophenyl)phosphine can generate a stable photoinduced luminescent radical in the crystalline state.^32^ Moreover, systematic investigations and in-depth understanding of the structure–property relationship of these triarylphosphine radicals remain unexplored.

**Fig. 1:**
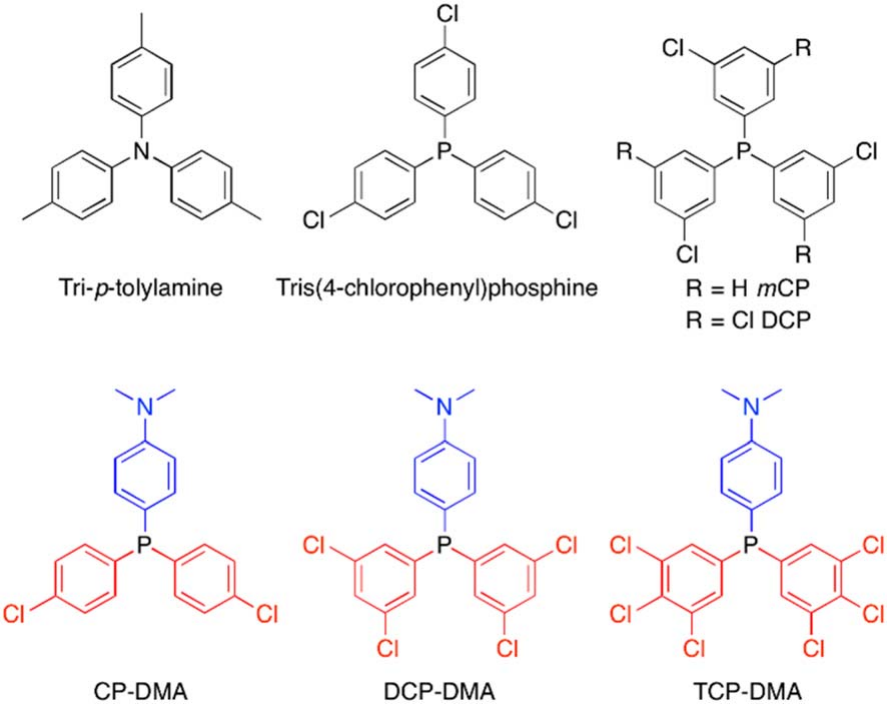
Selected photoinduced radical examples of tri-*p*-tolylamine^15^ and tris(4-chlorophenyl)phosphine^32^ and investigated molecular structures of chlorinated triphenylphosphines.

Herein, we report a series of donor–acceptor (D–A) structured triarylphosphines displaying interesting photochromism through photoinduced formation of stable radicals in the crystalline state. A photoinduced radical of D–A structured triarylphosphines was proved to be quite stable, and a significant radical signal persists up to several months when stored under ambient conditions. As shown in Fig. 1, by using *N*,*N*-dimethylaniline as the donor and different substituted chlorophenylphosphines as the acceptor, three triarylphosphine derivatives named CP-DMA, DCP-DMA, and TCP-DMA are designed. In-depth investigations revealed the structure–property relationship. The intrinsic D–A molecular structure breaks the peripheral symmetric architecture, allows photoinduced intermolecular electron transfer among the molecular chains formed by D–A counterpart pairing, and thus results in the formation of radical ion pairs. The higher stability of the radical cation ensures its long survival, which is beneficial for the photochromic performance. Besides, more chlorines on the acceptor increase the intramolecular charge-transfer character and also the oxidation potential. However, too strong D–A interaction is harmful to maintain the continuous intermolecular chains, and thus decreases the extent of photoinduced-radical generation. The current study provides a valuable insight into the stable organic radicals based on triarylphosphines in the solid-state.

## Results and discussion

**Scheme 1:**
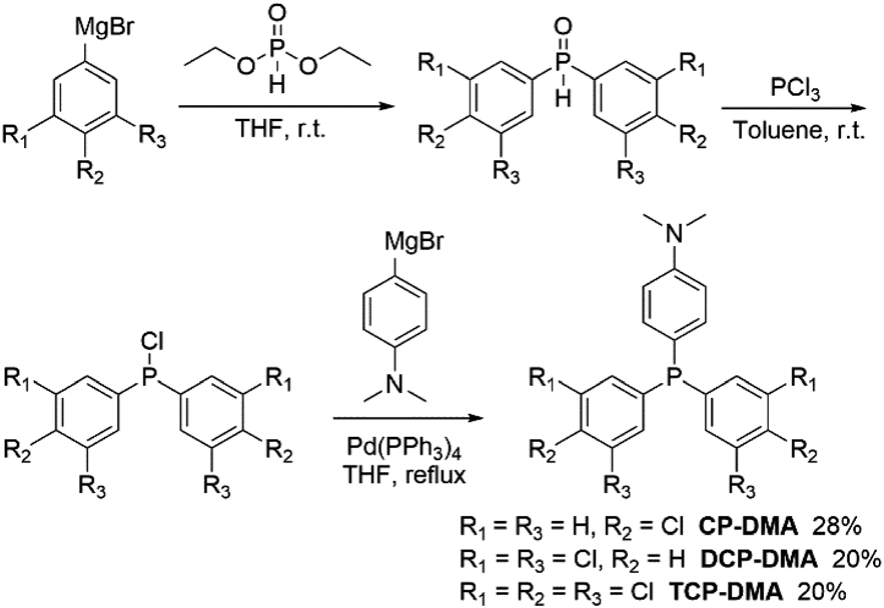
Synthetic routes of D–A type chlorinated triphenylphosphines.

Three symmetric phosphines were included for comparison (Scheme S1†). Tris(4-chlorophenyl)phosphine^32^ and tris(3-chlorophenyl)phosphine (*m*CP) were purchased and purified by column chromatography and recrystallization from ethanol and hexane three times prior to use. DCP is synthesized by one-pot cross-coupling of phosphorous trichloride with a freshly prepared Grignard reagent (Scheme S2†).^33^ The detailed synthetic routes of CP-DMA, DCP-DMA, and TCP-DMA are depicted in Schemes 1 and S3–S5.† The phosphorous chlorides are reduced from phosphine oxides and cross coupled with 4-(*N*,*N*-dimethyl)aniline magnesium bromide by Pd(PPh_3_)_4_. Three D–A type triarylphosphines of CP-DMA, DCP-DMA, and TCP-DMA are obtained with moderate overall yields. The products were characterized by ^1^H and ^13^C NMR spectroscopy (Charts S1–S8†), high-resolution mass spectrometry (Charts S12–S16†), differential scanning calorimetry, thermogravimetric analysis (Charts S17–S21†), cyclic voltammetry (Charts S22 and S23†) and single-crystal X-ray diffraction analysis (Tables S1–S5†).

**Fig. 2:**
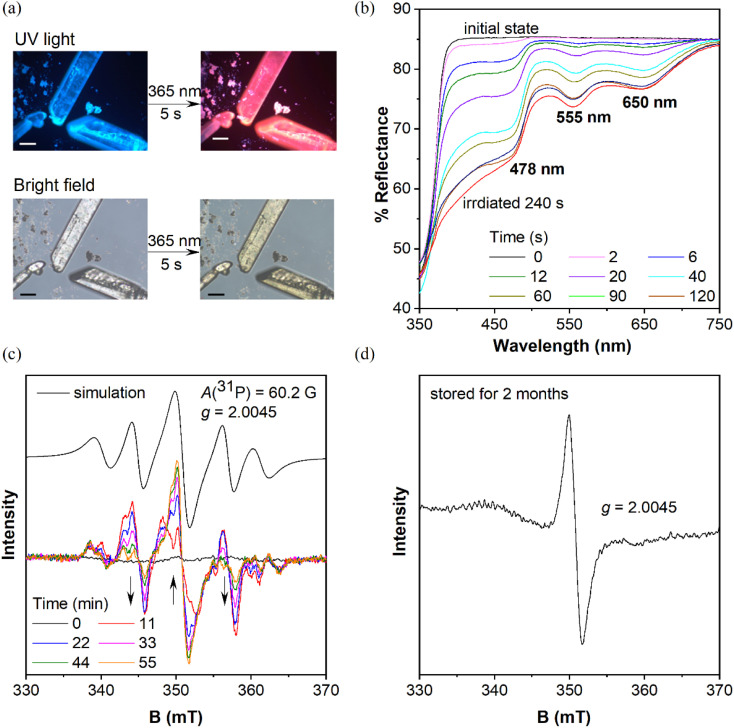
(a) Top: photographs of CP-DMA crystals from the fluorescence microscope before (left) and after (right) UV irradiation (340–390 nm) for 5 seconds; bottom: photographs of CP-DMA crystals from the fluorescence microscope in room light before (left) and after (right) UV irradiation (340–390 nm) for 5 seconds; scale bar = 50 μm. (b) Changes in the UV-vis reflectance spectra of the CP-DMA crystals upon irradiation at 365 nm (150 mW cm^−2^). (c) Simulation and time-dependent EPR spectra of CP-DMA crystals upon photoirradiation; (d) EPR spectra of photoirradiated CP-DMA crystals stored for two months.

When these purified triphenylphosphines are irradiated under UV light at 365 nm (150 mW cm^−2^), two symmetric chlorinated phosphines of *m*CP and DCP remain unchanged after 5 min irradiation (Fig. S1†), which was different from the reported photochromic tris(4-chlorophenyl)-phosphine.^15^ So, only chlorine substitution on triphenylphosphine is not essential for photochromism. In contrast, CP-DMA, DCP-DMA, and TCP-DMA with the D–A structure exhibited different photo-responsive color changes. The CP-DMA crystal exhibited blue fluorescence under UV light. After irradiation with an LED UV lamp (365 nm, 150 mW cm^−2^) under ambient conditions for 5 s, its emission color changed from blue to pink immediately (Fig. 2a, Video S1†). Besides, the appearance of CP-DMA crystals changed from colorless to brown-yellow. DCP-DMA demonstrated a similar photochromic effect but at a much lower rate. Upon irradiation with an LED UV lamp (365 nm, 150 mW cm^−2^) under ambient conditions for 5 min, the fluorescence and bright-field images of the DCP-DMA crystal changed from blue to slightly pink and from colorless to slightly yellow (Fig. S2†), respectively. The colorless TCP-DMA crystal did not change appearance and fluorescence upon UV irradiation for 5 min (Fig. S3†). These results suggested that less chlorine substituents were beneficial but not essential for the photochromic properties of triphenylphosphine.

Considering that the phosphine skeleton is electron-rich and easily oxidized, we irradiated CP-DMA and DCP-DMA crystals in a glove box under anhydrous and deoxygenated conditions (oxygen <0.1 ppm, water <0.1 ppm). The same color changes were observed immediately upon photo-triggering (Fig. S4†), which excluded the possibility of triarylphosphine photooxidation by environmental oxygen. To further confirm this, we oxidized CP-DMA to its oxide form by H_2_O_2_, which is utilized as the standard for comparison (Charts S9–S11†). The oxide product appeared as a white solid and showed blue emission under UV, which is not the same emission color as the photo-irradiated CP-DMA (Fig. S5†). To gain a deeper insight into the photochromic process, the crystalline samples before and after UV irradiation were subjected to powder X-ray diffraction, Fourier transform infrared spectroscopy, and NMR analyses, and no discernable change was observed (Charts S24–S26†), which eliminated the possibilities of other photoreactions and morphology changes. Besides, the purity of the CP-DMA molecule is confirmed by HPLC (Chart S27†), because a trace amount of impurity may influence the luminescence properties greatly which are not found. Afterwards, we employed UV-vis reflectance spectroscopy to monitor the photoinduced color change of CP-DMA crystals (Fig. 2b). Before UV irradiation, CP-DMA crystals exhibit no absorption in the visible light region. Upon irradiation at 365 nm, three strong absorption band peaks at 478 nm, 555 nm, and 650 nm emerged and their intensity increased as the irradiation time was prolonged. The response was obvious and fast in the beginning, then slowed down and was saturated after 1.5 min (Fig. S6†). Additionally, UV-vis reflectance spectra of DCP-DMA showed absorbance bands at wavelengths 480 nm and 565 nm, respectively, similar to CP-DMA (Fig. S7†). TCP-DMA crystals didn't show any changes upon continuous UV irradiation (Fig. S8†), which is consistent with the observed appearance.

To understand the origin of the species after photoirradiation, electron paramagnetic resonance (EPR) spectra of triply recrystallized CP-DMA crystals (5.9 mg) were obtained (Fig. 2c). The EPR spectrum was a flatline for colorless crystals before irradiation. Upon irradiation with UV light, several EPR signals appeared with a central *g* value of 2.0045, which was consistent with the value of the free electron of 2.0023.^34–36^ Time dependent EPR spectra showed the details of signal evolution and hyperfine splitting (Fig. 2c). Two sets of hyperfine couplings in the complicated EPR spectra of the irradiated CP-DMA crystal were found and quite difficult to fully resolve. In particular, as the irradiation was prolonged, the central signal for the free radical was gradually enhanced, while the two nearby peaks were decreased (Fig. S9†), which suggests that more than one intermediate radical was generated in the initial state and some were short-lived. Simulating the overall EPR spectrum provides a *g* value of 2.0045 and hyperfine coupling constant of 60.2 Gauss (Table S7†), which fell in the range of persistent phosphorus radical cations and radical anions.^23–26^ The *A*(^31^P) value indicated that the unpaired electron was not fully localized on the phosphorus atom as in traditional phosphorus radicals.^27,28,37^

Interestingly, an EPR signal could be detected from these irradiated CP-DMA crystals even after two months (Fig. 2d). The spectrum became simple with only one central mono-radical resonance at *g* = 2.0045, demonstrating the decay of radicals and fading away from the phosphorus center after long time storage. The stored samples could regenerate multiple complex EPR signals after being re-irradiated for a few minutes, switching the emission color from pink to red. Besides, the EPR spectra of the DCP-DMA crystal exhibit a broadened signal with even ten unresolved hyperfine components (Fig. S17†). These features are similar to those of the radical cation and radical anion pairs reported earlier by Wang^38^ and Braunschweig.^39^ Nevertheless, these results unequivocally revealed the radical-involved and complicated nature of the photochromic process for these sensitive triphenylphosphines.

**Fig. 3:**
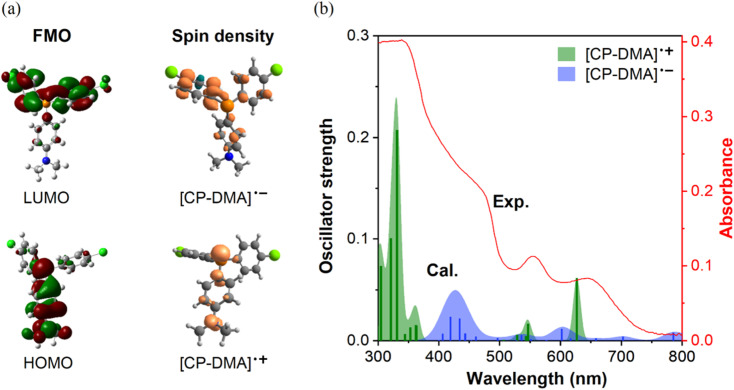
(a) Left: frontier molecular orbitals (FMOs) of CP-DMA using DFT with the UB3LYP/6-31+G(d,p) basis set, right: calculated spin density of the CP-DMA radical cation and CP-DMA radical anion in the gaseous state. (b) Comparison of experimental and calculated UV-vis spectra. Data of experimental UV-vis spectra came from the CP-DMA crystal after 240 s irradiation as shown in Fig. 2b. The bars are the oscillator strengths of the CP-DMA radical cation (green) and CP-DMA radical anion (blue).

As is well known, photoinduced intermolecular electron-transfer could initiate the radical ion pair, that is, the radical cation and radical anion, from neutral molecules.^40–42^ Considering the molecular D–A structure, we speculated that radicals originated from the photoinduced single electron transfer process. To test our hypothesis, density functional theory (DFT) and time-dependent DFT calculations on the proposed radical cation and anion were performed using the UB3LYP/6-31+G(d,p) function. In neutral CP-DMA, the electron cloud distribution of the HOMO mainly localized on the donor moiety of *N*,*N*-dimethylaniline and the phosphonium core and that of the LUMO spread over the chlorinated phenyl rings (Fig. 3a), supporting its charge transfer characteristics. The calculated spin density plot of the radical cation and radical anion matched well with the electron distribution on the original HOMO and LUMO, respectively. The reason is that the electron-rich part of the molecules donated the HOMO and stabilized the radical cation to form the SOMO, while the electron-poor part of the molecules contributed the LUMO and stabilized the radical anion to form the SOMO. Besides, the calculated spin density mapping illustrated that the spin density spread over the *N*,*N*-dimethylaniline group and phosphorus atom in the radical cation. The quantitative values were estimated as 39% at the phosphorus atom, 23% at the nitrogen atom, and 38% over the phenyl rings, which was in accordance with the simulated EPR spectra. Besides, the absorption shapes and intensities of the proposed radical cation and anion were calculated in terms of distinct oscillator strength (*f*) and transition contribution. As displayed in Fig. 3b and S10,† the radical cation [CP-DMA]˙^+^ exhibited two main peaks at 546 nm (*f* = 0.0165) and 625 nm (*f* = 0.0615) in the visible region, which were attributed to the excitations to S_3_ (with 51% SOMO − 3 → SOMO contribution) and S_2_ transitions (with 99% SOMO − 2 → SOMO contribution), respectively. Radical anion [CP-DMA]˙^−^ contributed several transitions in the visible region, including the main band between 400 and 500 nm. Remarkably, merging the calculated absorption peaks of the radical anion and cation was in good agreement with the total UV-vis reflectance spectra of irradiated CP-DMA crystals. Therefore, we further believed that the photochromism originated from the photoinduced radical ion pair in the initial state.

The radical anion and radical cation with different stabilities could decay gradually through multiple possible channels, including reacting with oxygen in the atmosphere,^43–46^ back electron-transfer to neutral molecules,^47,48^ eliminating halogen in the anion^49,50^ and reorganizing radical molecular structures,^51^*etc.* These complexities should be associated with different evolution trends in the time-dependent EPR spectra. From the experimental results, the radical cation was predicted with higher stability and to be long lived due to the easily accessible delocalization over the *N*,*N*-dimethylaniline subgroup, while the radical anion was more reactive and decayed quickly due to the poor stabilization from the peripheral molecular structure, easy oxidation in atmosphere and possible chlorine atom elimination. As these processes occurred in the solid state, single crystals of *m*CP, DCP, CP-DMA, DCP-DMA, and TCP-DMA suitable for XRD analysis were grown from their ethanol or petroleum ether solutions (Tables S1–S5†).

**Fig. 4:**
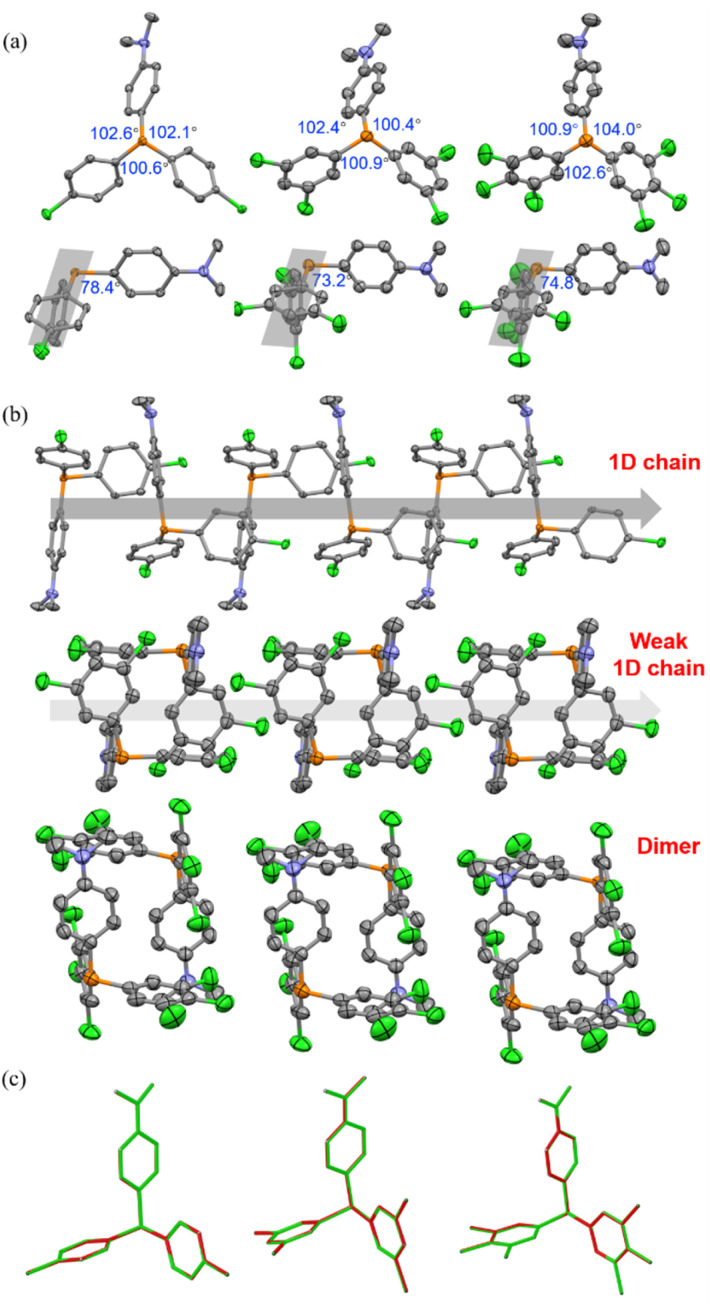
(a) Top: molecular conformations and selected angles of CP-DMA, DCP-DMA, and TCP-DMA in the crystalline state with thermal ellipsoids at 50% probability. Hydrogen atoms are omitted for clarity. Bottom: the angles between the average plane of the two acceptor phenyl rings and the donor DMA ring in the CP-DMA, DCP-DMA, and TCP-DMA single crystals. (b) Crystal packing diagrams of CP-DMA, DCP-DMA, and TCP-DMA. (c) Overlay single-crystal structures of CP-DMA, DCP-DMA, and TCP-DMA before (green skeleton) and after (red skeleton) UV irradiation for 10 min.

As depicted in Fig. 4a and S14,† in the crystalline state, *m*CP and DCP molecules maintained the quasi *C*_3_ symmetry, while CP-DMA, DCP-DMA, and TCP-DMA molecules endow the peripheral architectures with slightly different ∠C–P–C angles between the neighboring phenyl groups. These CP-DMA, DCP-DMA, and TCP-DMA phosphines intrinsically break the *C*_3_ symmetry by D–A counterpart alternation. Previously reported photochromic tris(4-chlorophenyl)-phosphine also broke the *C*_3_ symmetry *via* a different mechanism of adopting occasionally molecular deformation during crystal packing. It suggested that the symmetry breaking of triarylphosphine should be a prerequisite for the photoinduced radicals and color change. Thus, constructing the D–A structure in triarylphosphine should be a sensible and practical design principle to achieving photoinduced radicals. Besides, *N*,*N*-dimethylaniline subgroups had large angles towards the average planes of chlorinated phenylenes (grey shadows), ranging from 73.2 to 78.4 degrees and indicating the poor π-conjugation between the donor and acceptor parts. This was in agreement with the separated HOMO and LUMO distributions (Fig. S19†) and the short wavelength (<350 nm) of UV-vis absorption (Fig. S11–S13†).

We further analyzed the molecular packing of CP-DMA, DCP-DMA, and TCP-DMA crystals (Fig. 4b and S15†). CP-DMA adopted a monoclinic crystal structure and a *P*2_1_/*c* space group. It formed a 1D chain with adjacent molecules *via* weak C–H⋯C interactions. Interestingly, electron donor subgroups of *N*,*N*-dimethylaniline were located closely to the corner of the electron acceptor part of 4-chlorophenylenes, alternatively, to form the 1D chain. It provided a good channel for intermolecular electron-transfer upon photoirradiation. In contrast, DCP-DMA and TCP-DMA crystallized in the triclinic space group of *P*1̄. The intermolecular 1D chain was gradually broken by forming closer dimers *via* multiple C–Cl⋯H and C–H⋯C interactions. Although *N*,*N*-dimethylaniline subgroups were still located closely to the corner of chlorinated phenylenes in these dimers, breaking the 1D chain destroyed electron-transfer channels within the crystals. This packing difference was reflected in the gradually weakened color change and poor radical generation upon photoirradiation from CP-DMA, to DCP-DMA, and to TCP-DMA. When CP-DMA and DCP-DMA were doped into a PVA polymer matrix at a low concentration of 1 wt%, no remarkable change was observed after UV irradiation for 30 min (Fig. S16†). The generation of radical ion pairs should be disrupted because phosphines are molecularly dispersed in the PVA matrix, and thus continuous 1D chains are fully broken.

Afterwards, we analysed the identical crystals before and after UV irradiation with single-crystal XRD. The structures of CP-DMA, DCP-DMA, and TCP-DMA before (green) and after (red) UV irradiation were overlaid (Fig. 4c). Alternation of red and green indicated good overlapping, while green or red alone represented poor overlapping. Although the changes are not significant, subtle molecular deformations are still observed. When chlorinated phenylenes were settled with good overlap, *N*,*N*-dimethylaniline has evidently deviate from each other in CP-DMA and DCP-DMA. The detailed dihedral angle and bond length also proved the subtle structural changes after photoirradiation (Table S6†). However, the changes are not significant compared to those of the reported phosphine radical cations,^24–26^ which suggested that a small portion of the molecules were transformed into radicals.

Then, we employed quantitative EPR measurements to determine the absolute number of spins based on the “Spin-Count” function in the Xepr software of a Bruker E500 Spectrometer. Accurate mass and EPR parameters were recorded (Table S8†). Then the number of spins is divided by the Avogadro constant to estimated the reactive radical persentage, which is, around 10^−4^ molar ratio. The results indicated that the photoinduced radical generation was quite inefficient, accounting for the subtle single-crystal structure changes before and after UV irradiation. The trend was further verified by the enhanced absolute number of spins from tris(4-chlorophenyl)phosphine to CP-DMA and DCP-DMA, which suggested that constructing the D–A structure was a practical way to achieve photoinduced phosphine radicals. To have a deep insight into the radical concentrations, we use TEMPO as an external standard to quantify the amounts of photoinduced radicals. As seen in Fig. S18,† 3.5 mg of CP-DMA generated the same amounts of radicals as 10 μL of a 1.3 mM TEMPO solution, suggesting that CP-DMA can form appreciable amounts of radicals with 0.14% concentration. It represents remarkably high quantity of about 1 in 715 molecules containing a radical, compared to reported examples. Similar calculations for DCP-DMA and *p*CP radicals were of a similar magnitude, with 0.06% and 0.03%, respectively. Further details of the EPR experiment and methods used to estimate the radical concentration can be found in the ESI †(Fig. S18, Table S9).

## Conclusions

Color change, EPR spectra, and quantitative spin counting proved that multiple chlorinations at the electron acceptor counterpart lowered the ability of forming photoinduced radicals. Crystal packing analysis depicted that more chlorine atoms had additional C–Cl⋯H interactions and formed isolated dimers by D–A^⋯^D–A pairing in crystals. Closely interacting dimers broke the continuous 1D channel that was beneficial for charge separation and radical stabilization and allowed quick back electron-transfer to annihilate radical ion pairs. Besides, multiple chlorinations increased the intramolecular charge-transfer effect due to the electron deficient characteristics of chlorine atoms. This was verified by separated HOMO–LUMO distributions and decreased energy gaps (Fig. S19†), red-shifted and solvent-dependent UV-vis absorption (Fig. S11–S13†), and increased oxidation potentials in cyclic voltammetry (Charts S22–S23†). The potentials were estimated to be 0.83 V for CP-DMA, 0.97 V for DCP-DMA, and 1.03 V for TCP-DMA, respectively, *versus* Fc^+^/Fc in dichloromethane. High oxidation potential increased the barrier of losing an electron and intermolecular electron-transfer. Finally, the calculated spin-density plots of the radical cations of CP-DMA, DCP-DMA and TCP-DMA indicated that multiple chlorinations decreased the contributions of the phosphorus atoms (Fig. S20†). Furthermore, the energy levels of SUMOs were deeply laid, which facilitated retaking the electrons and thus increased the instability of radical cations (Fig. S21–S23†).

In summary, we reported a series of D–A structured triarylphosphines and disclosed the noticeable photochromism upon photoirradiation in the crystalline state. Photoinduced organic radicals associated with central phosphorus and their evolution with time were carefully verified and proved to be the reason for the color change. X-ray crystallographic analysis combined with theoretical calculations revealed the intrinsic symmetry breaking in peripheral architecture, which formed continuous molecular chains by D–A counterpart pairing. Intermolecular electron-transfer occurred and resulted in radical ion pairs upon photo triggering. The current study depicts a fundamental understanding of achieving stable organic radicals based on triarylphosphine in the solid-state.

## Data availability

All necessary information is included in the ESI.†

## Author contributions

C. T. performed all experimental work and data analysis and wrote the manuscript. L. S. performed the DFT calculations. K. Z. and P. R. analyzed the XRD data. E. Z. corrected the manuscript. Z. H. supervised the project and corrected the manuscript.

## Conflicts of interest

There are no conflicts to declare.

## Supplementary Material

SC-014-D2SC05753K-s001

SC-014-D2SC05753K-s002

SC-014-D2SC05753K-s003
