# Supplementary material for: Manipulating D–A interaction to achieve stable photoinduced organic radicals in triphenylphosphine crystals
Source: Chem Sci. 2023 Jan 18;14(7):1871–7. doi: 10.1039/d2sc05753k (PMC9930928; doi:10.1039/d2sc05753k)
Supplement: SC-014-D2SC05753K-s001 [file SC-014-D2SC05753K-s001.pdf]

Electronic Supplementary Material (ESI) for Chemical Science.
This journal is © The Royal Society of Chemistry 2023

Manipulating D-A Interaction to Achieve Stable Photoinduced Organic Radicals in Triphenylphosphine Crystals

Chunlin Tang, [a+] Lijuan Song, [a+] Kang Zhou, [b] Peng Ren, [a] Engui Zhao [a] and Zikai He*[a,c]

[a] *School of Science, Harbin Institute of Technology, Shenzhen, Guangdong 518055, China.*

[b] *Hoffmann Institute of Advanced Materials, Shenzhen Polytechnic, Shenzhen, Guangdong 518055, China*

[c] *School of Chemical Engineering and Technology, Harbin Institute of Technology, Harbin, Heilongjiang 150001, China.*

[+] *These authors contributed equally to this work.*

* *Corresponding authors:* hezikai@hit.edu.cn

Table of Contents

Materials and methods S2
Synthesis and characterization S2
Photophysical properties S19
Single crystal data S25
EPR measurements........ S30
Computational details........ S32
References S42

Materials and methods

Materials: Chemicals and solvents were purchased from *Energy Chemical*, *J&K* Scientific Ltd., Bide Pharmatech Ltd. without further purification. Deuterated solvents were purchased from Bide Pharmatech Ltd.

Characterization methods: ^{1}H NMR and ^{13}C NMR spectra were collected on a Q.One Instruments Quantum-I 400 M. Tetramethylsilane was used as the internal standard. High-resolution mass spectrometry analyses were conducted on a Bruker FTMS. Single crystal X-ray diffraction (XRD) were performed on Bruker Smart APEXII CCD diffractometer using graphite monochromated CuKα or MoKα radiation. Cyclic voltammetry was performed on a CHI660E electrochemical workstation with platinum as the working electrode and Fc^+/Fc (0.1 M in CH_3CN) as the reference, and the measurement was carried out under a nitrogen atmosphere. ESR spectra were measured on an ELEXSYS-II E500 spectrometer (Bruker Co, Ltd, Germany, ~9.45 GHz) in X-range at room temperature. Differential scanning calorimetry (DSC) and Thermogravimetric analysis (TGA) were measured on a PE STA 8000 under air environment (heating rate: 10 °C/min). UV-vis spectra were measured on a SHIMADZU UV 2600 spectrophotometer.

Synthesis and characterization

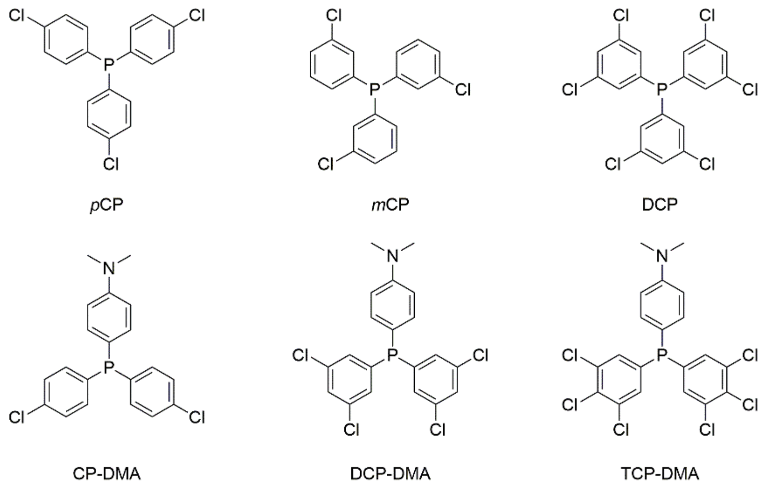

Scheme S1. The chemical structures of triaryl phosphines studied in the work.

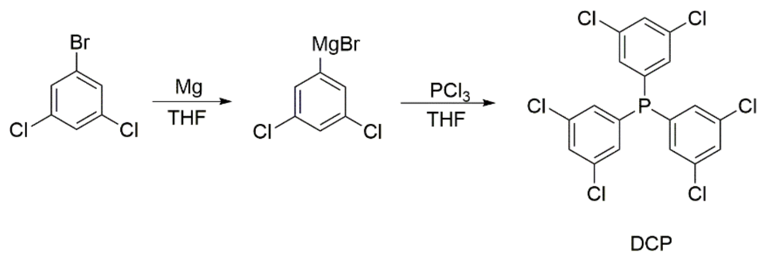

Scheme S2. The synthetic route of DCP.

*p*CP (tris(4-chlorophenyl)phosphine) and *m*CP were purchased from Energy Chemical Ltd. and purified by silica-gel column chromatography and recrystallization from ethanol and hexane at least three times. DCP was synthesized according to the procedures reported previously[1]. To a

suspension of magnesium turnings (864 mg, 36 mmol) in 15 mL of anhydrous THF, was added a solution of 1-bromo-3,5-dichlorobenzene (6.7 g, 30 mmol) in 15 mL of anhydrous THF dropwisely. The reaction started spontaneously after about 2-3 min, with obvious color change and raising temperature of the reaction mixture. The solution of the arylbromine was added continuously at a rate necessary to maintain reflux. After the addition was completed, the mixture of PCl_3 (5 mL, 2 M in DCM) in 20 mL of anhydrous THF was added at a rate necessary to maintain reflux. Afterwards, the mixture was stirred for one hour at ambient temperature. After completion of the reaction, 50 mL of water was added, and the organic layer was separated. The aqueous layer was extracted twice with 30 mL of diethyl ether. The combined organic phase was dried with magnesium sulfate. The solvent was evaporated under reduced pressure. The raw product was subjected to silica-gel column chromatography and then recrystallized from hot ethanol to obtain DCP as a white solid in a 28% yield. ^{1}H NMR (400 MHz, $CDCl_3$), δ (ppm): 7.42 (s, 3H, Ar-H), 7.12-7.10 (dd, J = 8.0 Hz, 4.0 Hz, 6H, Ar-H). ^{13}C NMR (100 MHz, $CDCl_3$) δ (ppm): 138.42, 138.24, 135.94, 135.86, 131.45, 131.24, 130.09. HRMS (ESI) *m/z* calcd. for $C_{18}H_9Cl_6NaP$ $[M+Na]^+$: 488.8465; found: 488.8508.

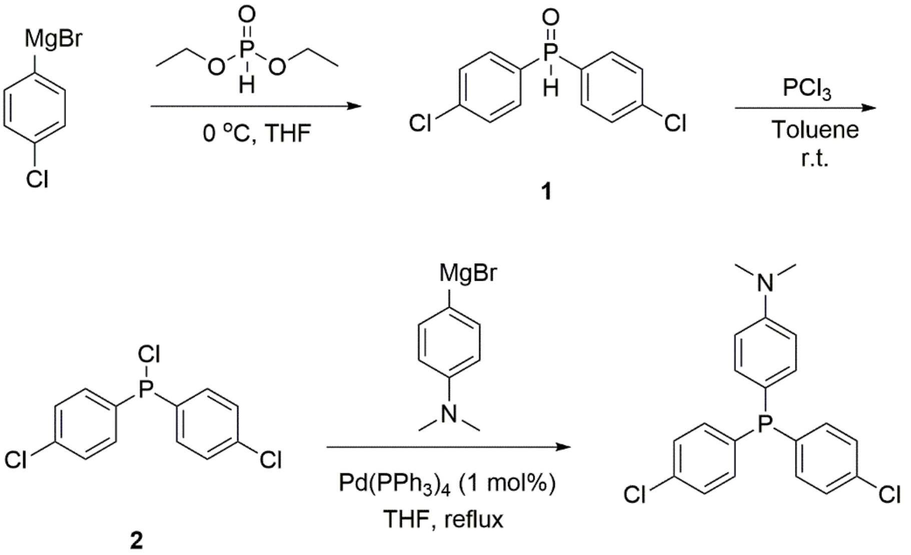

Scheme S3. The synthetic route of CP-DMA.

The procedure for the synthesis of CP-DMA:

1 was synthesized according to the procedures reported previously[2]. Under a nitrogen atmosphere, a solution of diethyl phosphite (2.6 mL, 20 mmol) in anhydrous THF (40 mL) was added slowly to a solution of Grignard reagents (60 mL, 60 mmol, 1 M in THF) at 0 °C. After stirring for an additional 5 h at room temperature, the reaction was quenched by ammonium chloride solution. The precipitated solid was removed by filtration. The filtrate was extracted with ethyl acetate, washed with saturated sodium chloride solution, and dried over $MgSO_4$. After filtration and concentration, pure product **1** was obtained by flash column chromatography on silica gel (PE/EA = 1:1). **2** was synthesized according to a known procedure[3]. To a stirring solution of PCl_3 (11.5 mmol, 1.0 mL, 1.0 eq) in toluene (15 mL) was added dropwisely diarylphosphine oxide **1** (11.5 mmol, 1.0 eq) in toluene (15 mL) at room temperature. The reaction mixture was stirred overnight. Distillation of the solvent under

vacuum gave the crude product **2**. A solution of $Pd(PPh_3)_4$ (0.1 mmol, 1%mol) in THF (5 mL) was added to the freshly synthesized (4-(dimethylamino)phenyl) magnesium bromide (10 mmol, 0.5 M in THF) and refluxed for 30 min. Then a solution of **2** (11.5 mmol, 1.1 eq) in THF was added slowly to the solution of (4-(dimethylamino)phenyl) magnesium bromide (10 mmol, 0.5 M in THF). The resulting mixture was allowed to refluxed at 80 °C for 4 hours and then quenched with saturated NH_4Cl solution, extracted with EA three times. The organic layer was dried with $MgSO_4$, concentrated by rotary evaporation. Further purification by flash column chromatography (PE/EA = 6/1) afforded CP-DMA (1.2 g, 3.2 mmol) as a white solid in 28% yield. ^{1}H NMR (400 MHz, $CDCl_3$), δ (ppm): 7.29-7.25 (m, 4H, Ar-H), 7.21-7.17 (t, J = 8.0 Hz, 6H, Ar-H), 6.70-6.68 (d, J = 8.0 Hz, 2H, Ar-H), 2.98 (s, 6H). ^{13}C NMR (100 MHz, $CDCl_3$) δ (ppm): 151.14, 137.07, 136.95, 135.65, 134.68, 134.62, 134.42, 128.70, 128.64, 112.44, 112.35, 40.20. HRMS (ESI) m/z calcd. for $C_{20}H_{19}Cl_2NP$ $[M+H]^+$: 374.0627; found: 374.0628.

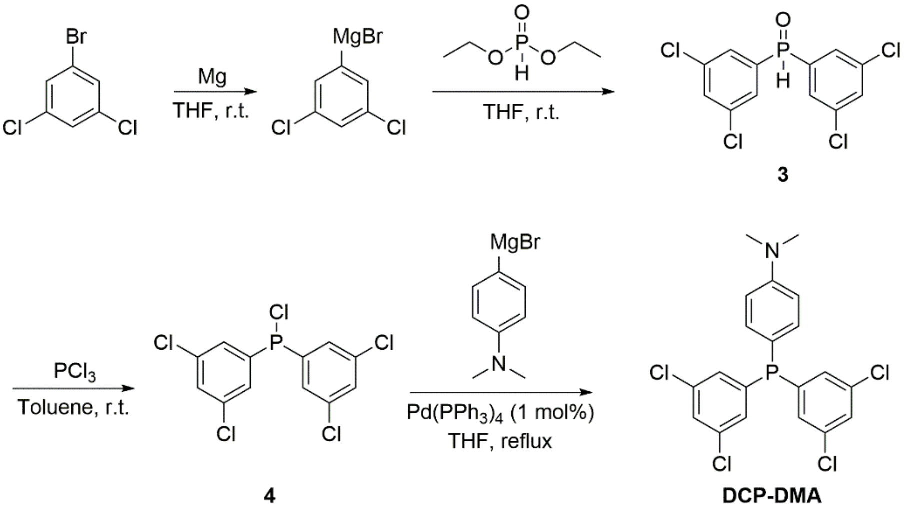

Scheme S4. The synthetic route of DCP-DMA.

The procedure for the synthesis of DCP-DMA:

Under a nitrogen atmosphere, a solution of diethyl phosphite (2.6 mL, 20 mmol) in anhydrous THF (40 mL) was added slowly to freshly synthesized Grignard reagents (60 mL, 60 mmol, 1M in THF) at 0 °C. After stirring for an additional 5 h at room temperature, the reaction was quenched by the ammonium chloride solution. The precipitated solid was removed by filtration. The filtrate was extracted with ethyl acetate, washed with saturated sodium chloride solution, and dried over $MgSO_4$. After filtration and concentration, pure product **3** was obtained by flash column chromatography on silica gel (PE/EA = 1:1). To a stiring solution of PCl_3 (11.5 mmol, 1.0 mL, 1.0 eq) in toluene (15 mL) was added dropwisely diarylphosphine oxide **3** (11.5 mmol, 1.0 eq) in toluene (15 mL) at room temperature. The reaction mixture was stirred overnight. Distillation of the solvent under vacuum gave the crude product **4**. A solution of $Pd(PPh_3)_4$ (0.1 mmol, 1 mol%) in THF (5 mL) was added to the freshly synthesized (4-(dimethylamino)phenyl) magnesium bromide (10 mmol, 0.5 M in THF) and refluxed for 30 min. Then, a solution of **4** (11.5 mmol, 1.1 eq) in THF was added slowly to the solution of (4-(dimethylamino)phenyl) magnesium bromide (10 mmol, 0.5 M in THF). The resulting mixture was allowed to reflux at 80 °C for 3 hours and then quenched with saturated NH_4Cl solution, extracted with EA three times. The organic layer was dried with $MgSO_4$, concentrated under reduced pressure. Further

purification by flash column chromatography (PE/EA = 6/1) afforded DCP-DMA (0.9 g, 2.0 mmol) as white solid in 20% yield.^{1}H NMR (400 MHz, $CDCl_3$), δ (ppm): 7.31 (s, 2H, Ar-H), 7.25-7.20 (m, 2H, Ar-H), 7.11-7.09 (dd, J = 8.0, 4.0 Hz, 4H, Ar-H), 6.73-6.71 (d, J = 8.0 Hz, 2H, Ar-H), 3.01 (s, 6H). ^{13}C NMR (100 MHz, $CDCl_3$) δ (ppm): 151.62, 142.29, 142.11, 136.06, 135.82, 135.42, 135.35, 131.07, 130.87, 128.84, 112.89, 112.50, 40.11. HRMS (ESI) m/z calcd for $C_{20}H_{17}Cl_4NP$ $[M+H]^+$: 443.9818; found: 443.9818.

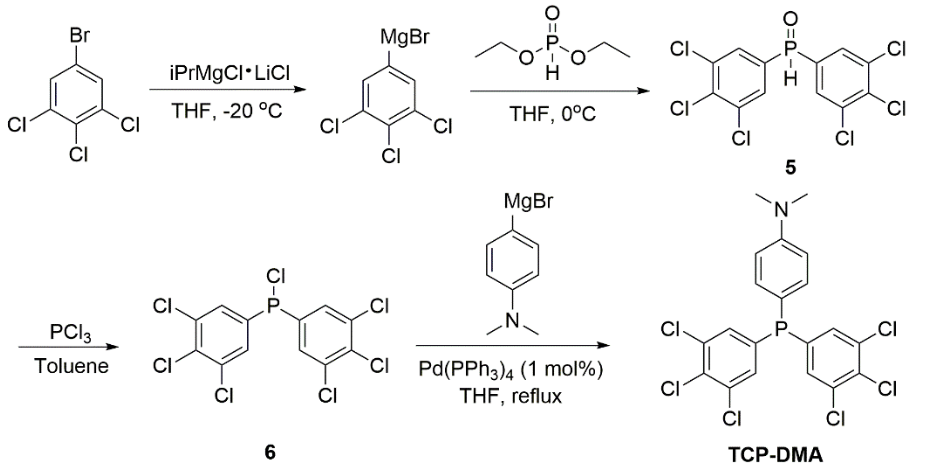

Scheme S5. The synthetic route of TCP-DMA.

The procedure for the synthesis of TCP-DMA:

To a stiring and cool (−20 °C) solution of 3,4,5-trichlorobromobenzene (15.6 g, 60 mmol) in anhydrous THF (40 mL) under nitrogen was added dropwisely *i*PrMgCl·LiCl (56 mL, 1.3 M in THF). The solution was stirred for 3 h at -20 °C. A solution of diethyl phosphite (2.3 mL, 18 mmol) in anhydrous THF (6 mL) was added slowly to freshly synthesized Grignard reagents (96 mL, 60 mmol, 0.6 M in THF) at 0 °C. After stirring for an additional 5 h at room temperature, the reaction was quenched by the ammonium chloride solution. The precipitated solid was removed by filtration. The filtrate was extracted with ethyl acetate, washed with saturated sodium chloride solution, and dried over $MgSO_4$. After filtration and concentration, pure product **5** was obtained by flash column chromatography on silica gel (PE/EA = 1:1). To a stirred solution of PCl_3 (11.5 mmol, 1.0 mL, 1.0 eq) in toluene (15 mL) was added dropwisely diarylphosphine oxide **5** (11.5 mmol, 1.0 eq) in toluene (15 mL) at room temperature. The reaction mixture was stirred overnight. Distillation of the solvent under vacuum gave the crude product **6**. A solution of $Pd(PPh_3)_4$ (0.1 mmol, 1 mol%) in THF (5 mL) was added to the freshly synthesized (4-(dimethylamino)phenyl) magnesium bromide (10 mmol, 0.5 M in THF) and refluxed for 30 min. Then a solution of **6** (11.5 mmol, 1.1 eq) in THF was added slowly to the solution of (4-(dimethylamino)phenyl) magnesium bromide (10 mmol, 0.5 M in THF). The resulting mixture was allowed to refluxed at 80 °C for 3 hours and then quenched with saturated NH_4Cl solution, extracted with EA three times. The organic layer was dried with $MgSO_4$, concentrated by rotary evaporation. Further purification by flash column chromatography (PE/EA = 6/1) afforded TCP-DMA (0.9 g, 2.0 mmol) as white solid in 20% yield.^{1}H NMR (400 MHz, DMSO-d_6), δ (ppm): 7.40-7.39 (d, J = 4.0 Hz, 4H, Ar-H), 7.27-7.23 (t, J = 8.0 Hz, 2H, Ar-H), 6.79-6.77 (d, J = 8.0 Hz, 2H, Ar-H), 2.95 (s, 6H). ^{13}C NMR (100 MHz,$CDCl_3$) δ (ppm): 151.74, 139.03, 138.84, 135.97, 135.74, 134.77, 134.70, 132.66, 132.45, 131.88, 112.68, 112.59, 40.11. HRMS (ESI) m/z calcd for $C_{20}H_{15}Cl_6NP$ $[M+H]^+$: 511.9038; found: 511.9041.

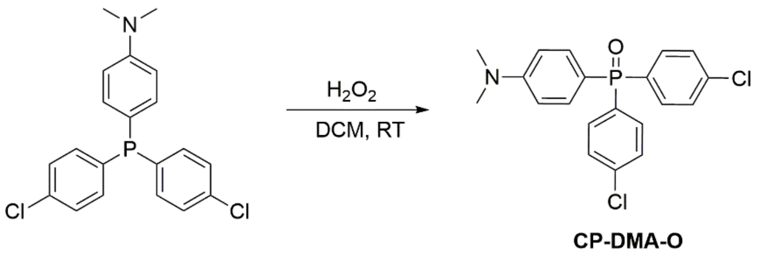

Scheme S5. The synthetic route of CP-DMA-O.

The procedure for the synthesis of CP-DMA-O:

To a round bottom flask equipped with a stir bar was added CP-DMA (46.1 mg, 0.12 mmol, 1.0 eq) and dichloromethane (3 mL). The resulting solution was cooled to 0 °C. Hydrogen peroxide (0.18 mmol, 30% aq, 1.5 eq.) was added to the round bottom. The reaction was stirred at RT until the complete disappearance of the starting material was observed by thinlayer chromatography (TLC). The reaction was diluted with DCM (10 mL) and water (5 mL). The organic layer was removed and dried over Na_2SO_4 and evaporated under reduced pressure to afford the crude product which was further purified by column chromatography using DCM/MeOH mixture as an eluent to give a white solid product (32.1 mg, 0.08 mmol) in 68% yield. ^{1}H NMR (400 MHz, $CDCl_3$), δ (ppm): 7.61-7.56 (dd, J = 6.0, 4.0 Hz, 4H, Ar-H), 7.43-7.40 (m, 6H, Ar-H), 6.74-6.72 (d, J = 8.0 Hz, 2H, Ar-H), 3.02 (s, 6H). ^{13}C NMR (100 MHz, $CDCl_3$) δ (ppm): 152.51, 138.36, 133.51, 133.40, 132.43, 131.39, 128.90, 128.77, 111.70, 111.56, 40.18. HRMS (ESI) m/z calcd. for $C_{20}H_{19}Cl_2NOP$ $[M+H]^+$: 390.0576; found: 390.0585.

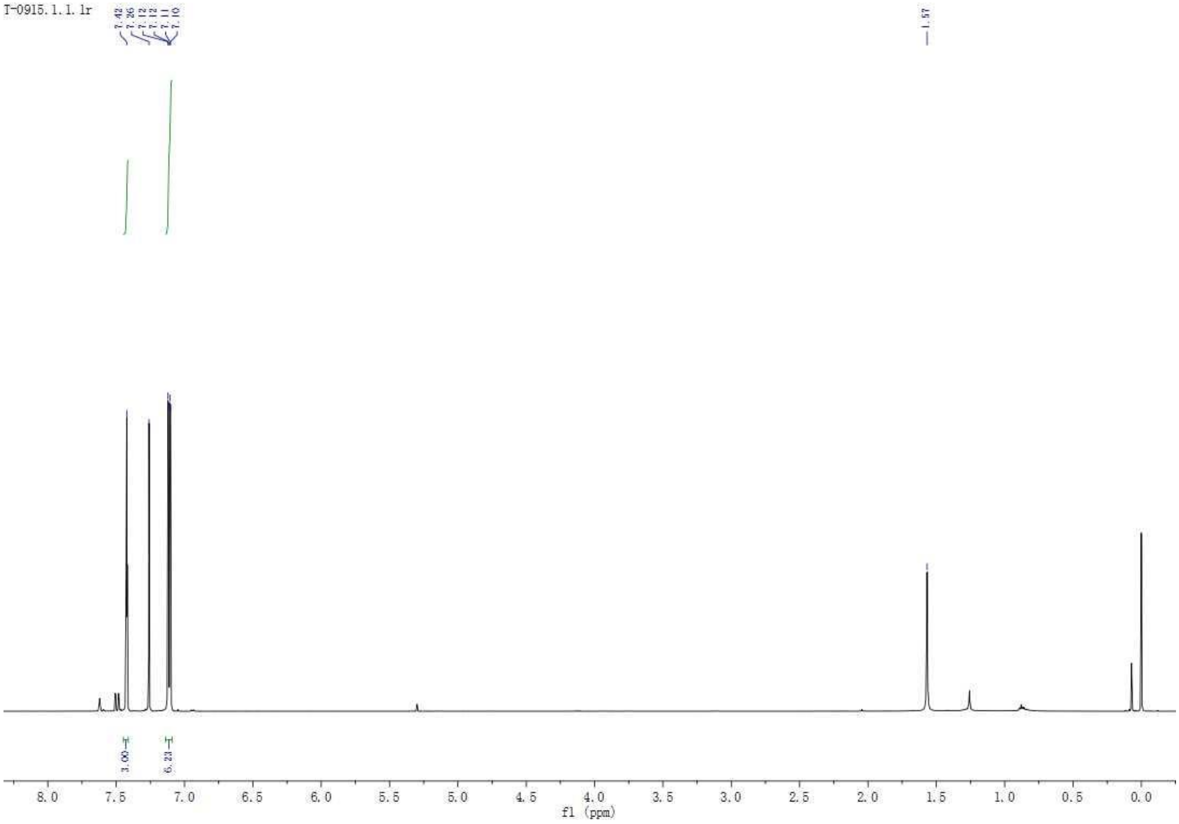

Chart S1. ^{1}H N MR spectrum of DCP in $CDCl_3$.

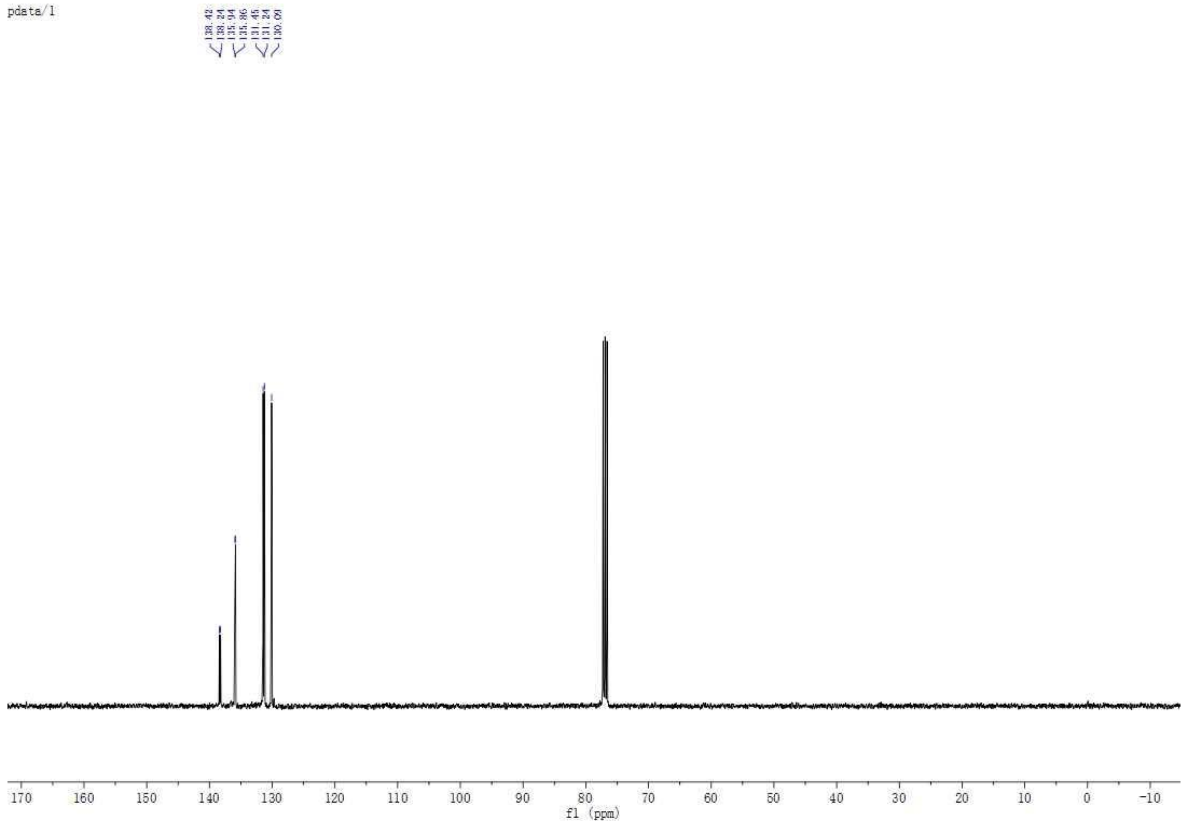

Chart S2. ^{13}C NMR spectrum of DCP in $CDCl_3$.

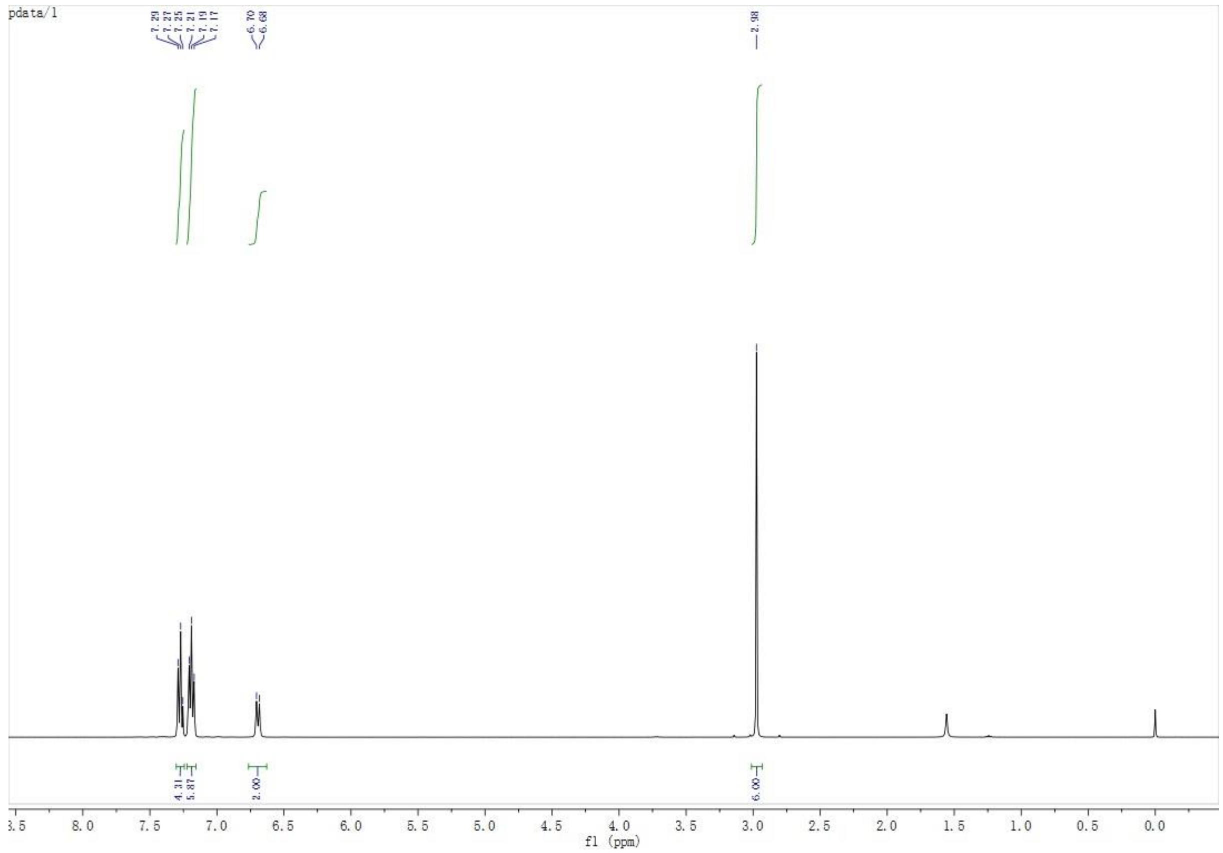

Chart S3. ^{1}H NMR spectrum of CP-DMA in $CDCl_3$.

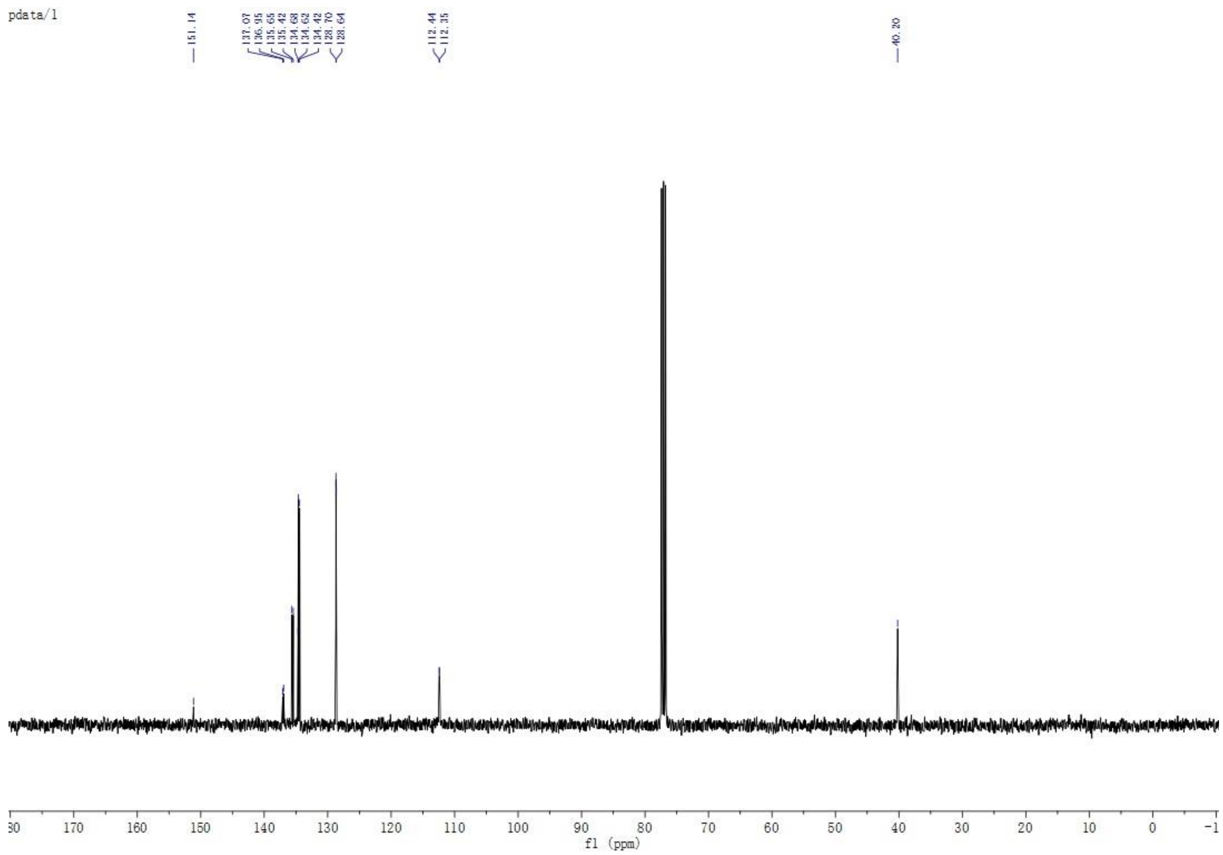

Chart S4. ^{13}C NMR spectrum of CP-DMA in $CDCl_3$.

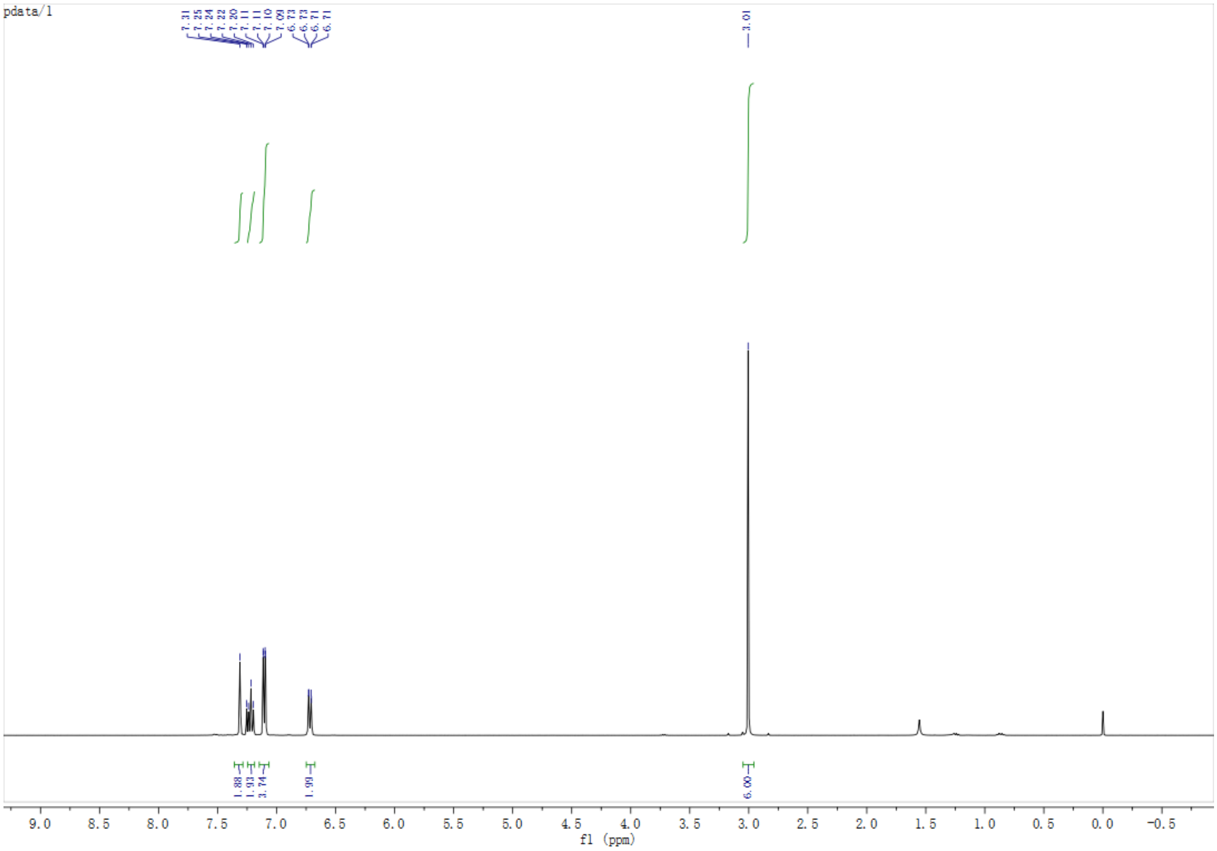

Chart S5. ^{1}H NMR spectrum of DCP-DMA in $CDCl_3$.

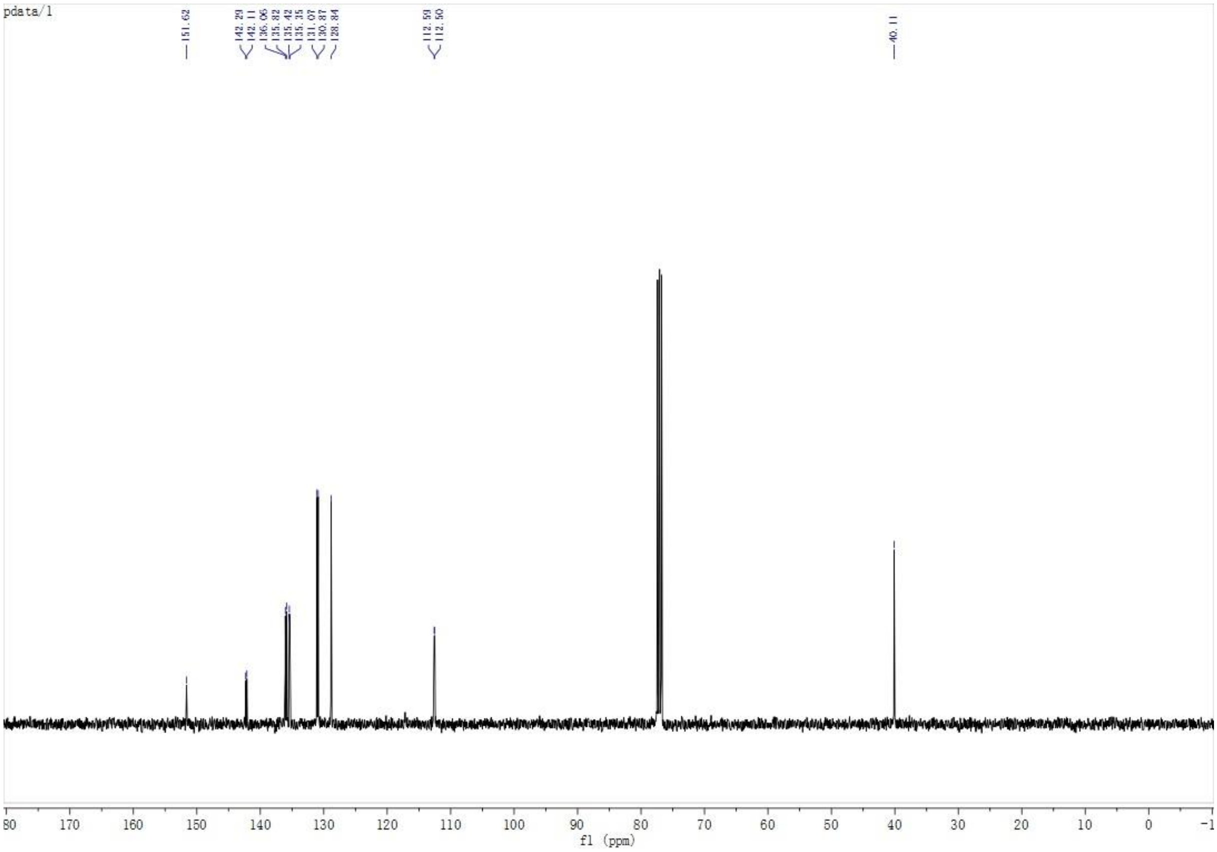

Chart S6. ^{13}C NMR spectrum of DCP-DMA in $CDCl_3$.

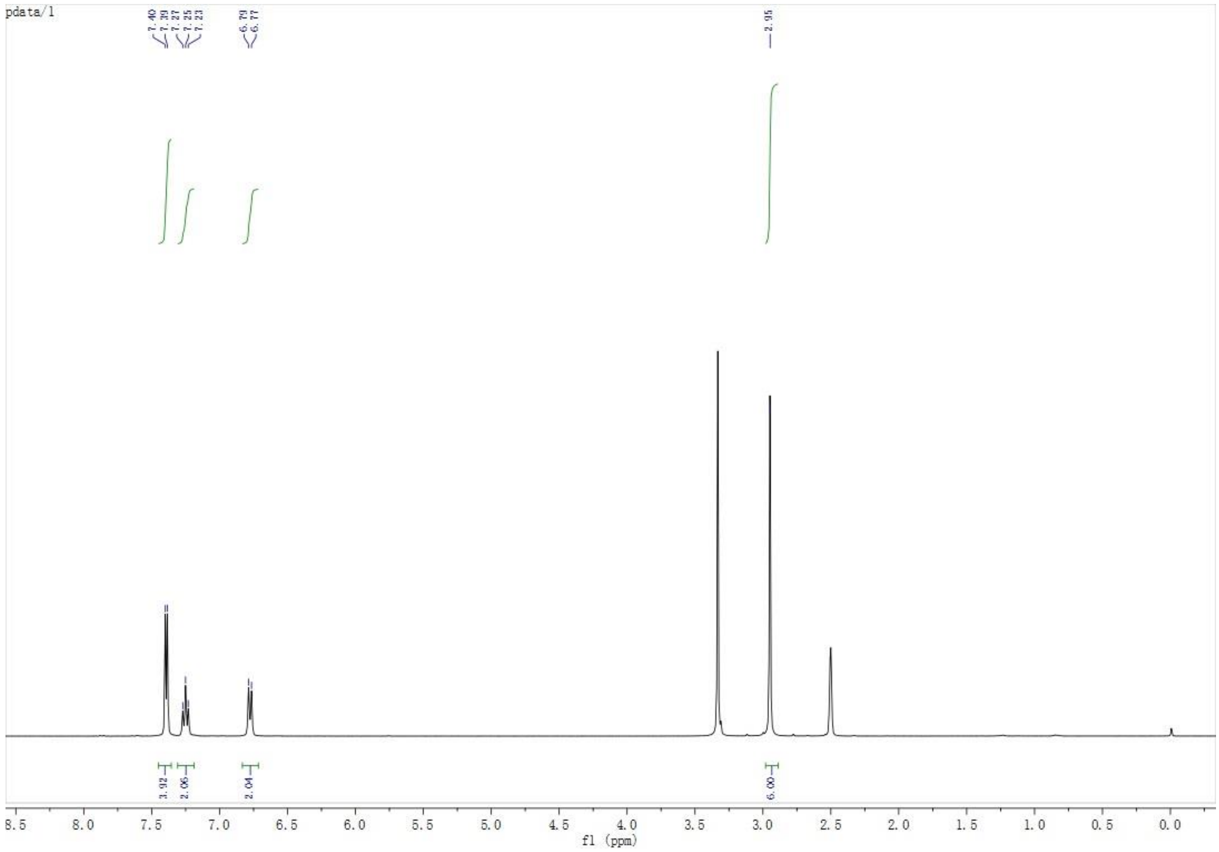

Chart S7. ^{1}H NMR spectrum of TCP-DMA in DMSO-*d*6.

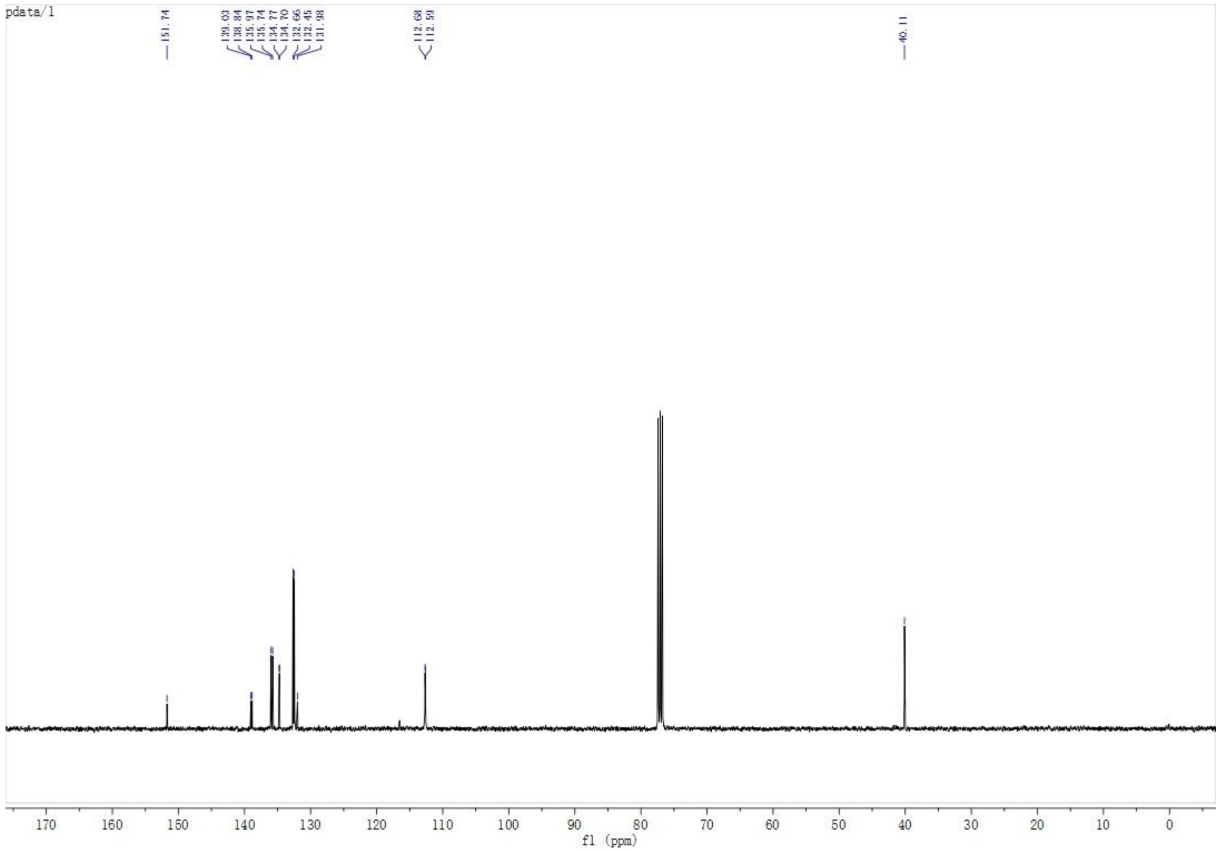

Chart S8. ^{13}C NMR spectrum of TCP-DMA in $CDCl_3$.

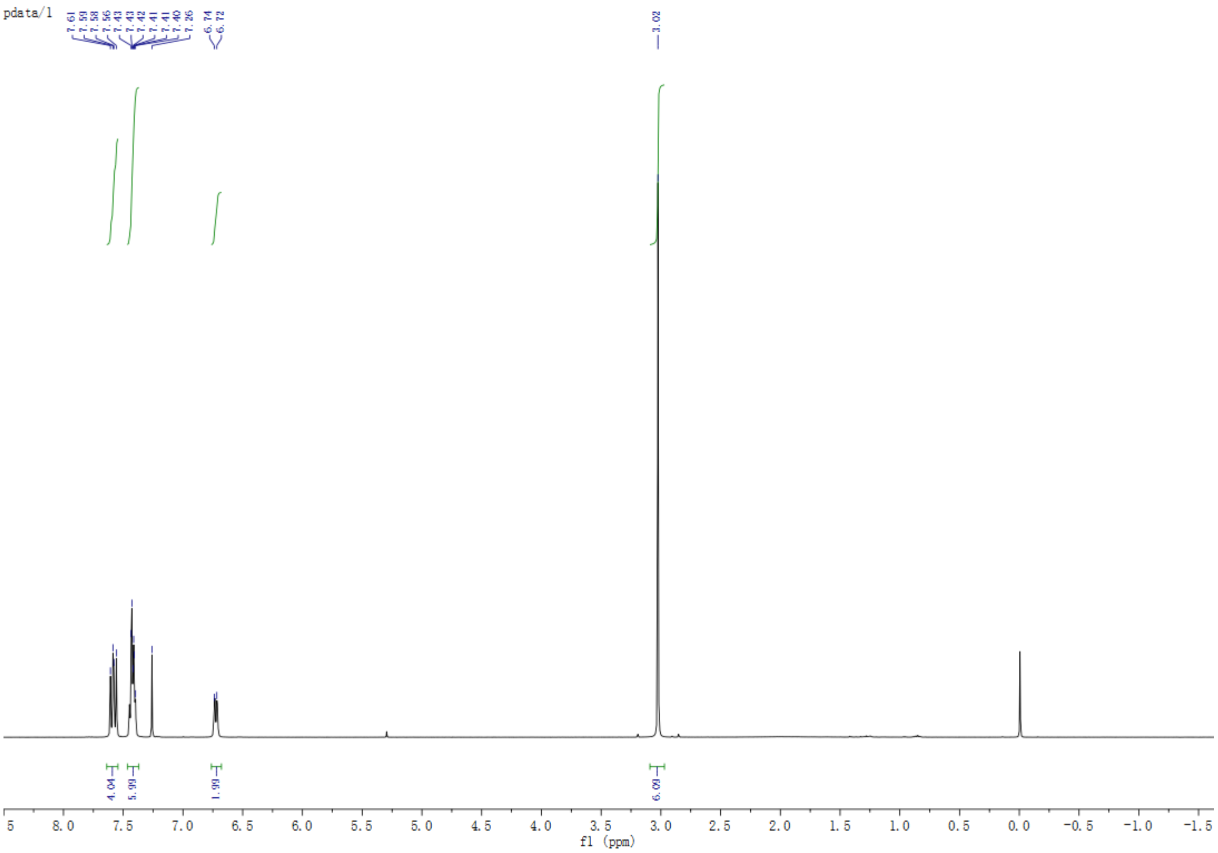

Chart S9. ^{1}H NMR spectrum of CP-DMA-O in $CDCl_3$.

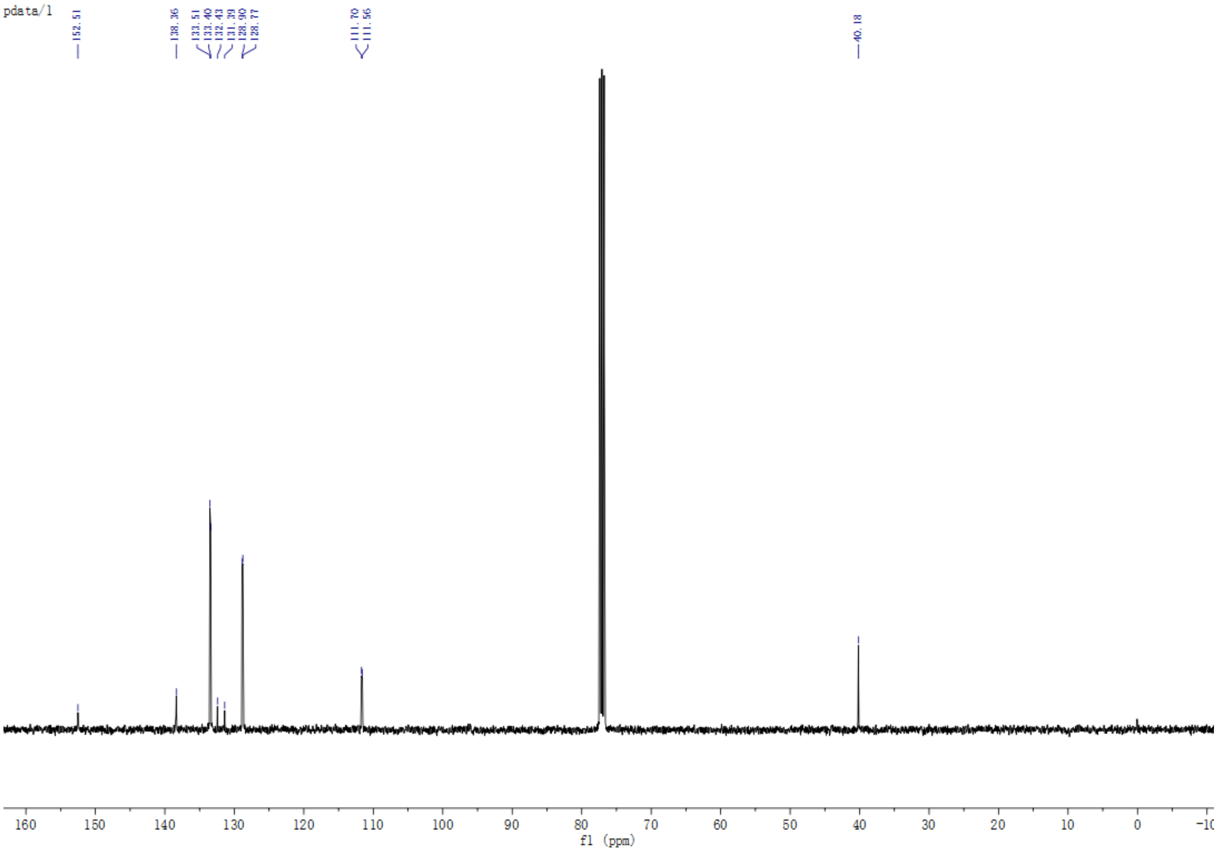

Chart S10. ^{13}C NMR spectrum of CP-DMA-O in $CDCl_3$.

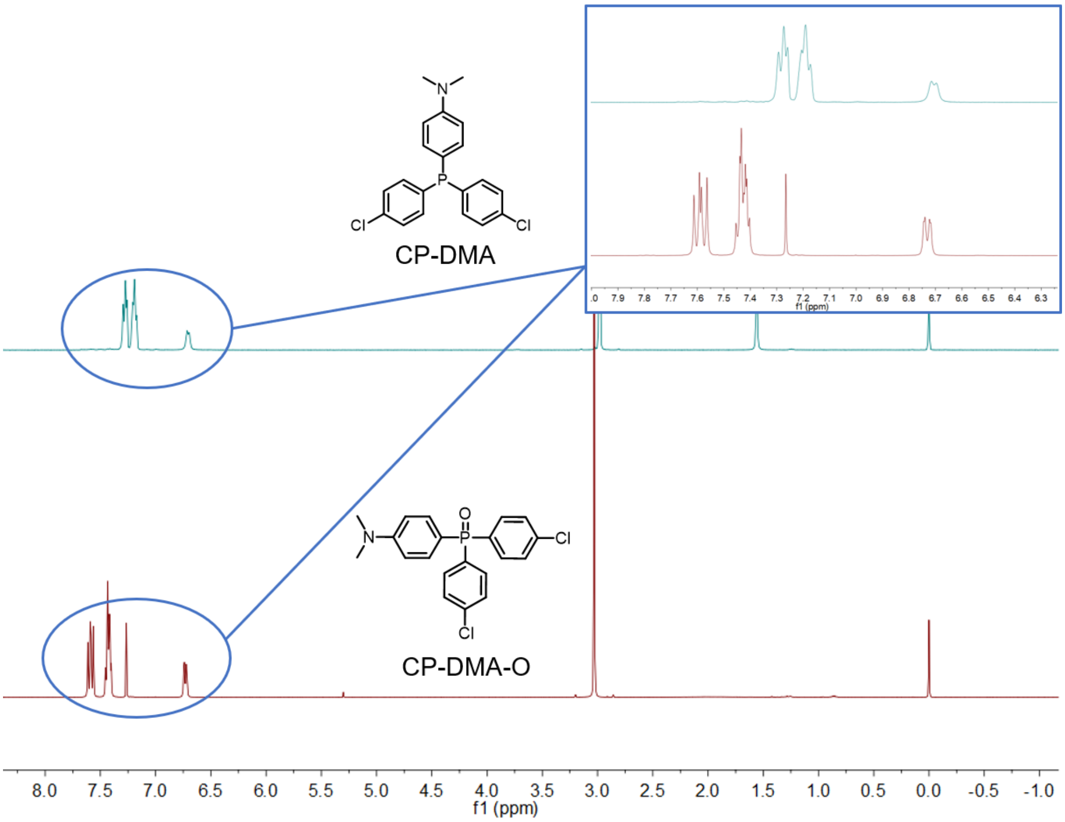

Chart S11. ^{1}H NMR spectra of CP-DMA and CP-DMA-O in $CDCl_3$ solution.

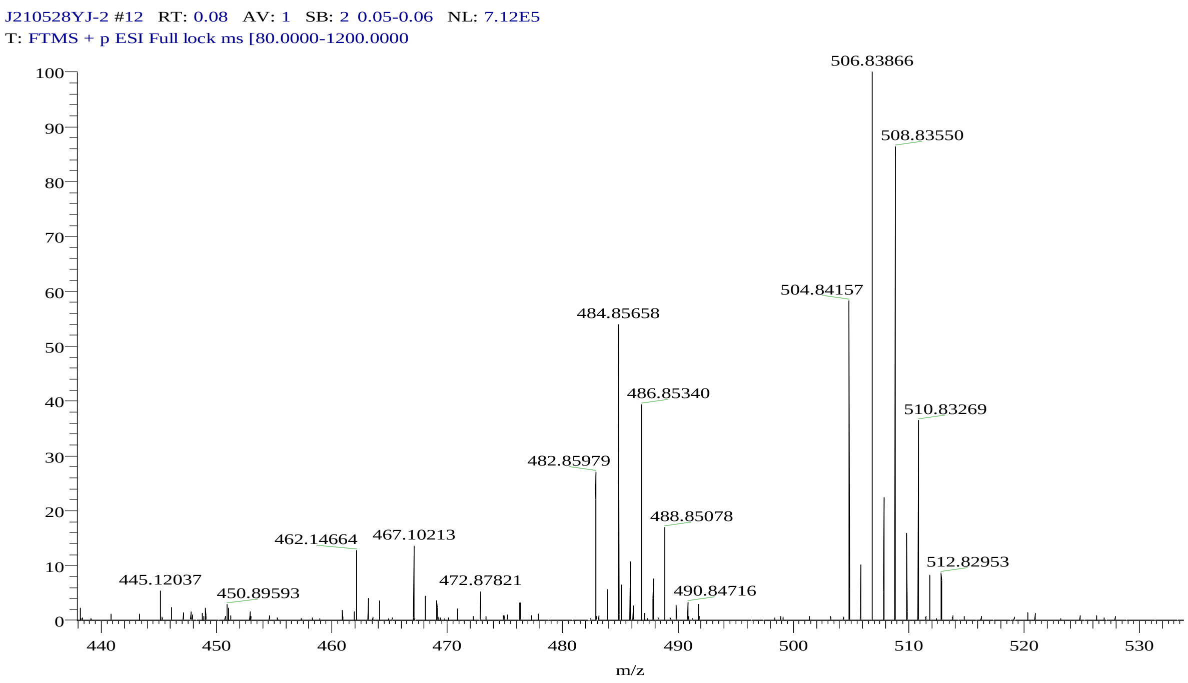

Chart S12. HRMS spectrum of DCP.

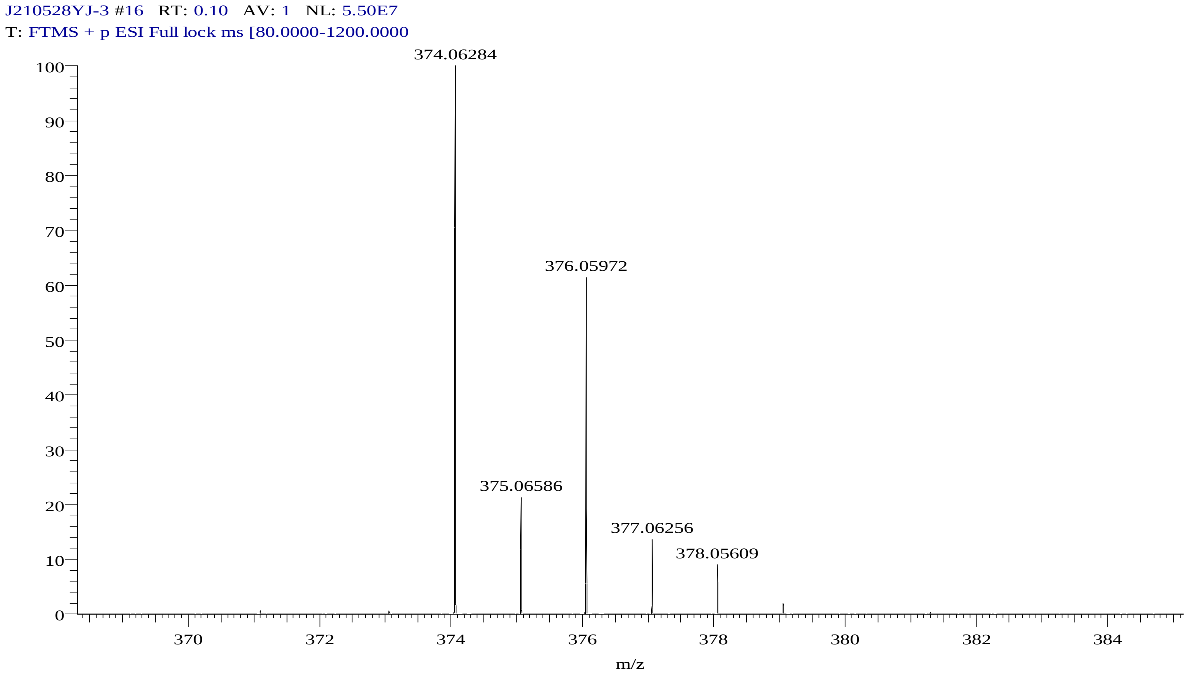

Chart S13. HRMS spectrum of CP-DMA.

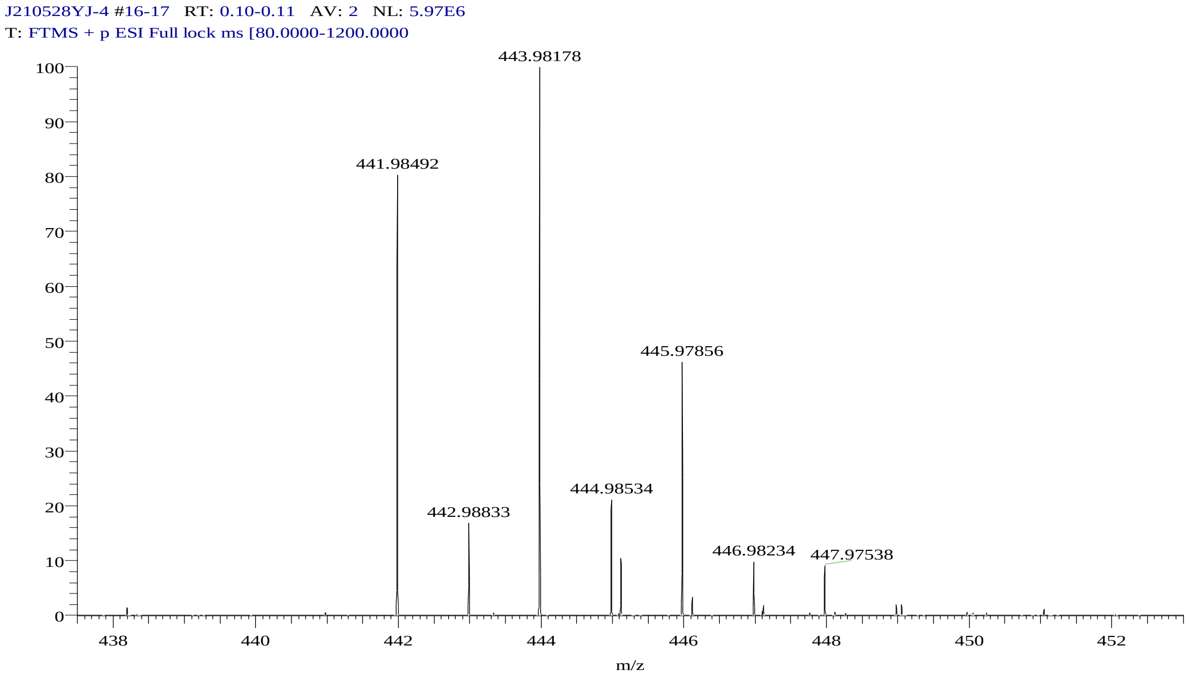

Chart S14. HRMS spectrum of DCP-DMA.

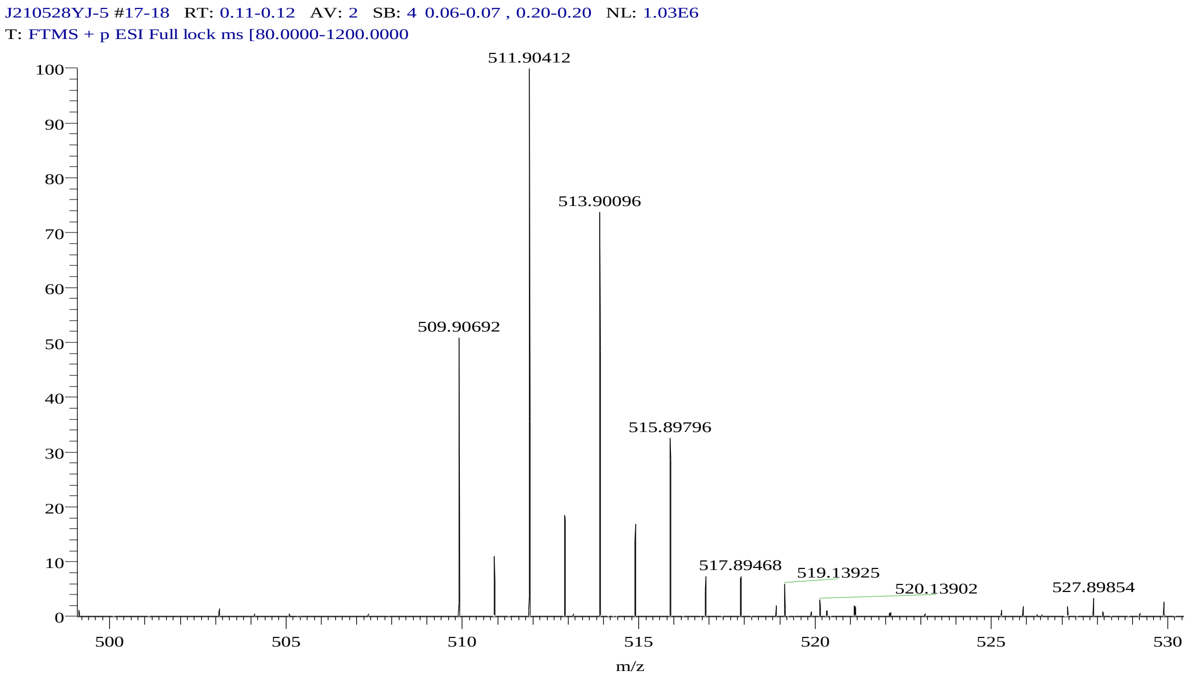

Chart S15. HRMS spectrum of TCP-DMA.

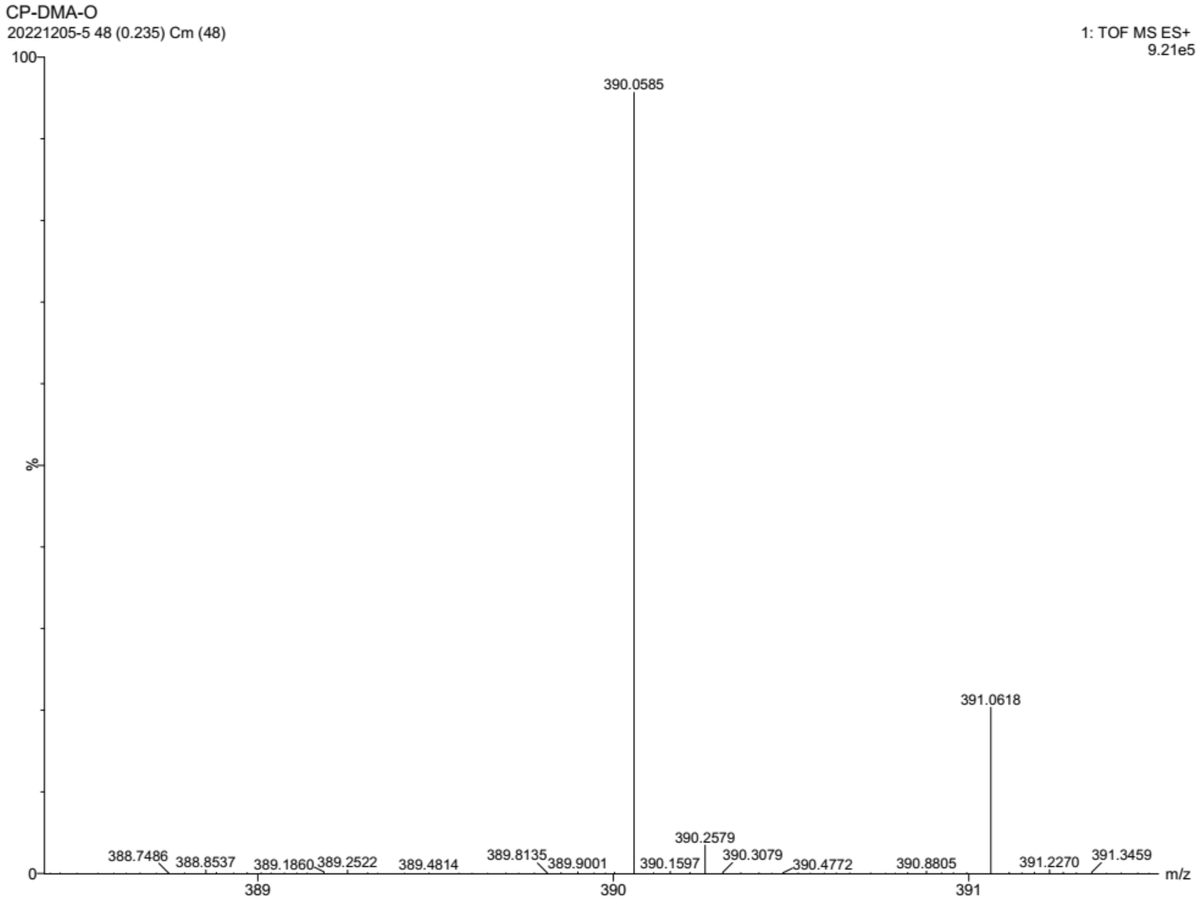

Chart S16. HRMS spectrum of CP-DMA-O.

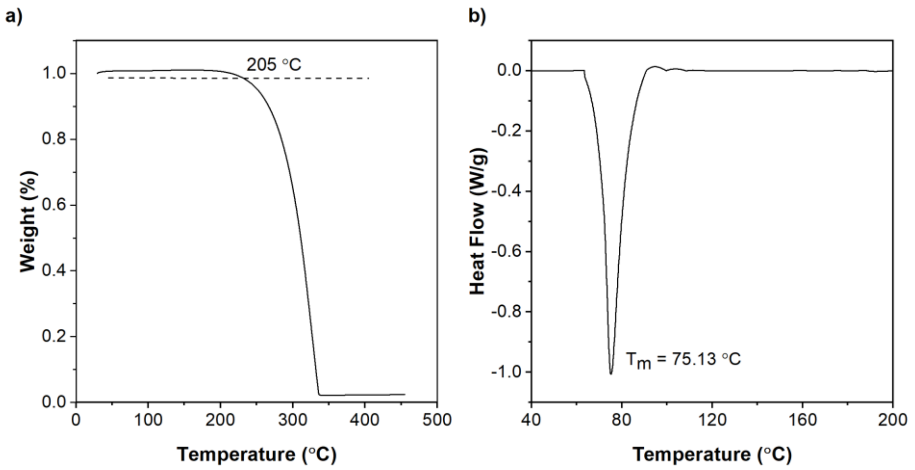

Chart S17. a) TGA curve and b) DSC thermogram of *m*CP. Both TGA and DSC were recorded under air at a heating rate of 10 °C/min.

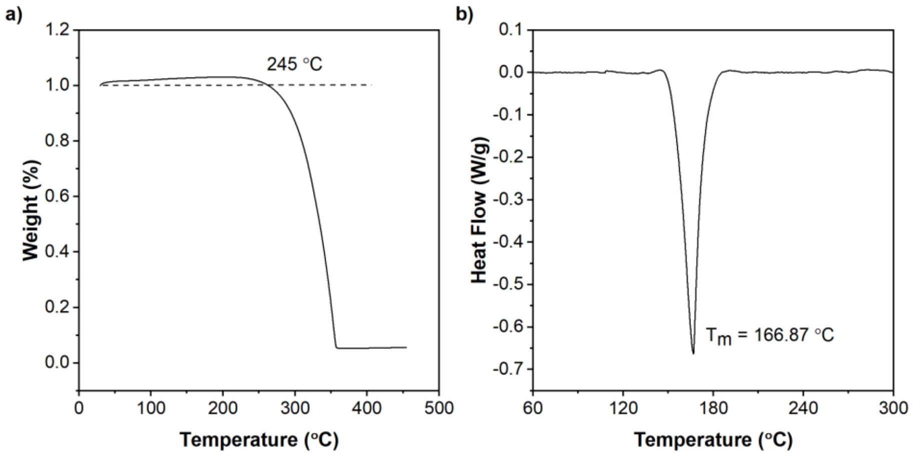

Chart S18. a) TGA curve and b) DSC thermogram of DCP. Both TGA and DSC were recorded under air at a heating rate of 10 °C/min.

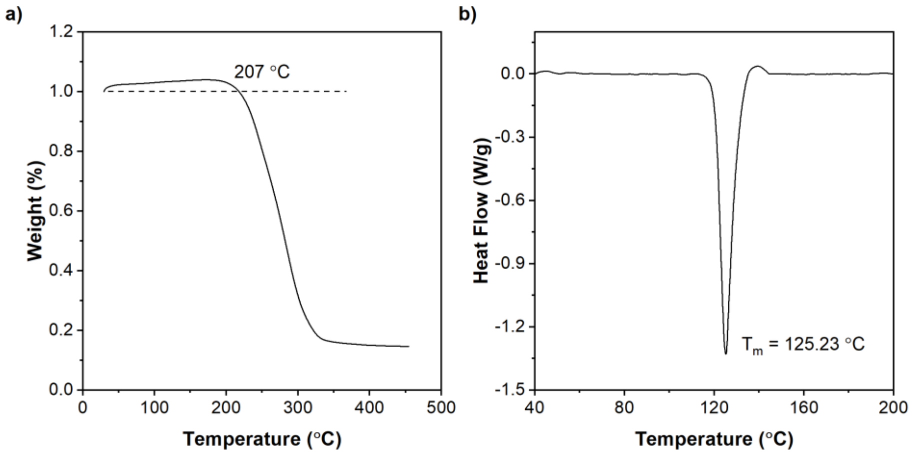

Chart S19. a) TGA curve and b) DSC thermogram of CP-DMA. Both TGA and DSC were recorded under air at a heating rate of 10 °C/min.

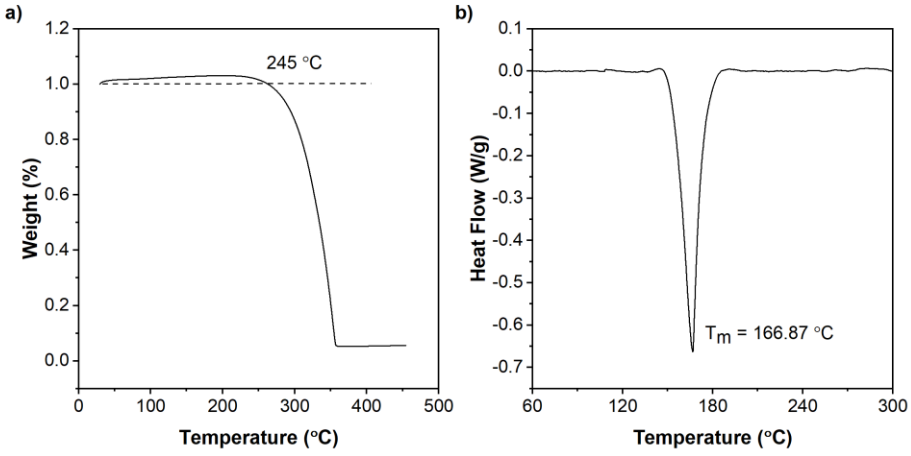

Chart S20. a) TGA curve and b) DSC thermogram of DCP-DMA. Both TGA and DSC were recorded under air at a heating rate of 10 °C/min.

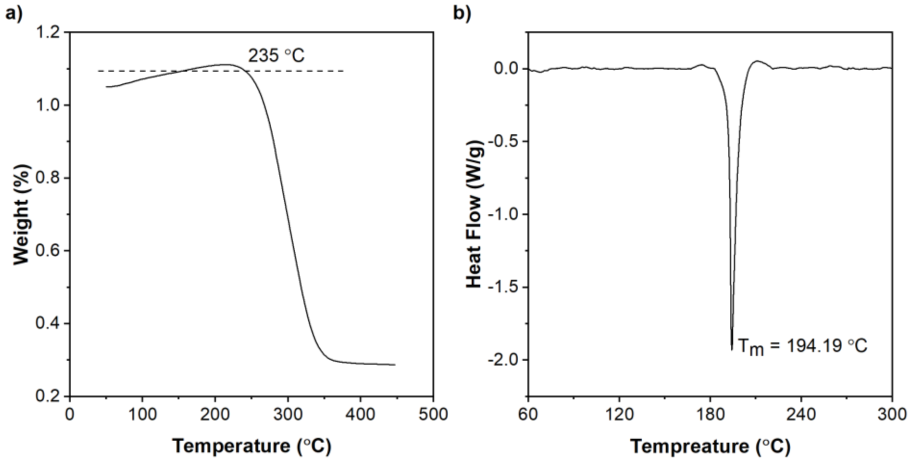

Chart S21. a) TGA curve and b) DSC thermogram of TCP-DMA. Both TGA and DSC were recorded under air at a heating rate of 10 °C/min.

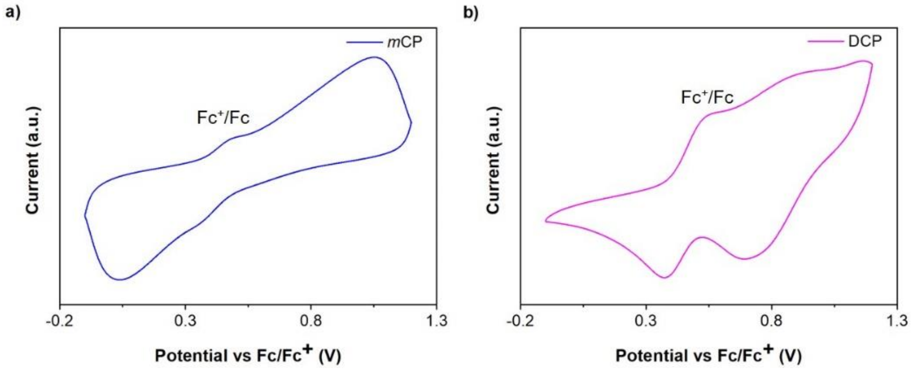

Chart S22. Cyclic voltammogram of *m*CP and DCP (1×10^{-3} M) in CH_2Cl_2, containing 0.1 M $^{n}Bu_4NPF_6$, measured at 100 mV s^{-1} at 25 °C.

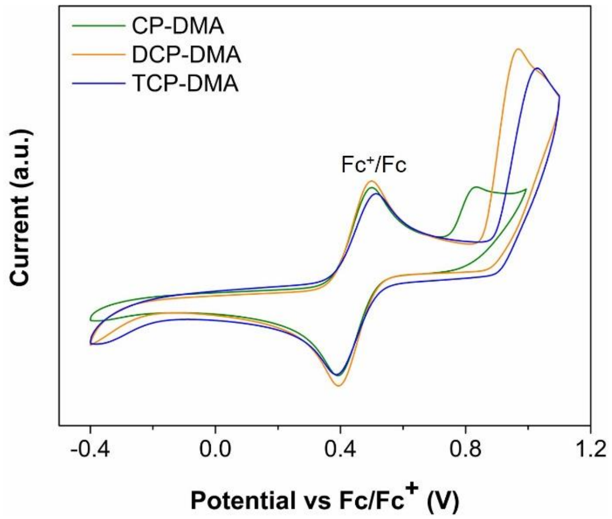

Chart S23. Cyclic voltammogram of CP-DMA, DCP-DMA, and TCP-DMA (1×10^{-3} M) in CH_2Cl_2, containing 0.1 M $^{n}Bu_4NPF_6$ (vs Fc^+/Fc), measured at 100 mV/s at 25 °C.

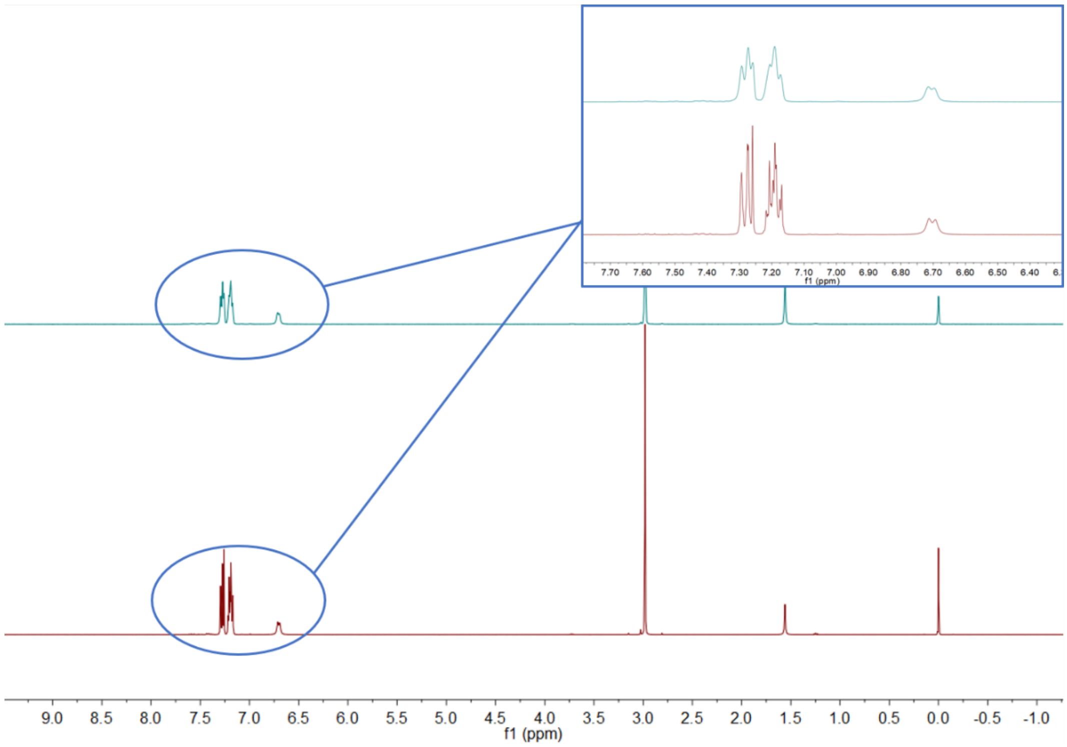

Chart S24. ^{1}H NMR spectra of CP-DMA in $CDCl_3$ solution before (up) and after (down) UV irradiation (5 min).

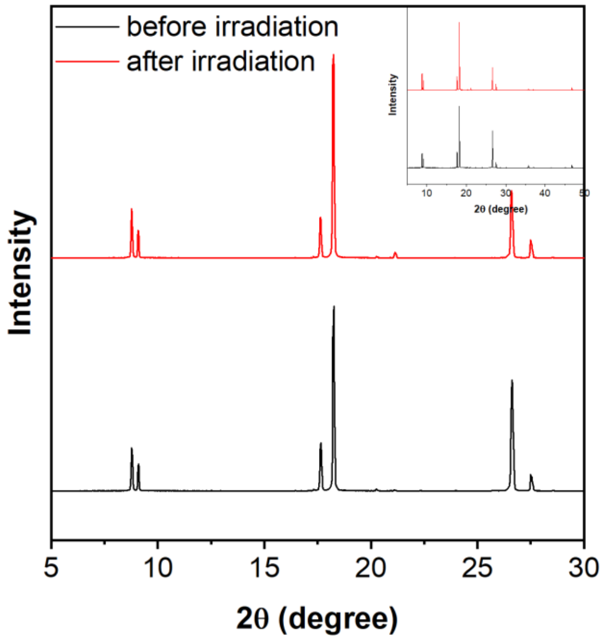

Chart S25. Powder XRD spectra of CP-DMA before and after irradiation (5 min).

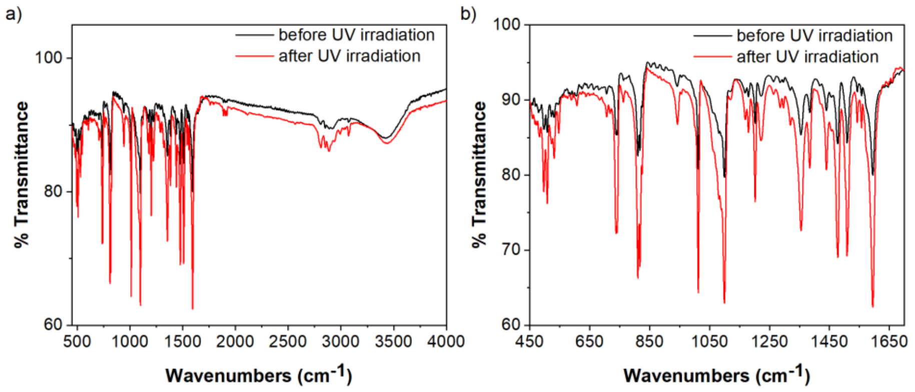

Chart S26. a) FTIR spectra of CP-DMA before and after UV irradiation (5 min). b) Local enlargement of FTIR spectra of CP-DMA from 450 cm^{-1} to 1700 cm^{-1} wavenumbers.

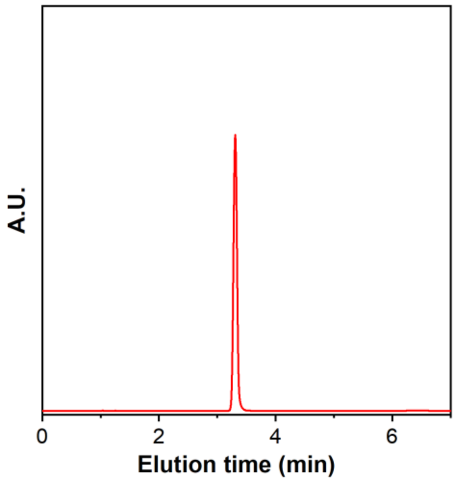

Chart S27. HPLC spectrum of CP-DMA molecules in 254 nm.

Photophysical properties

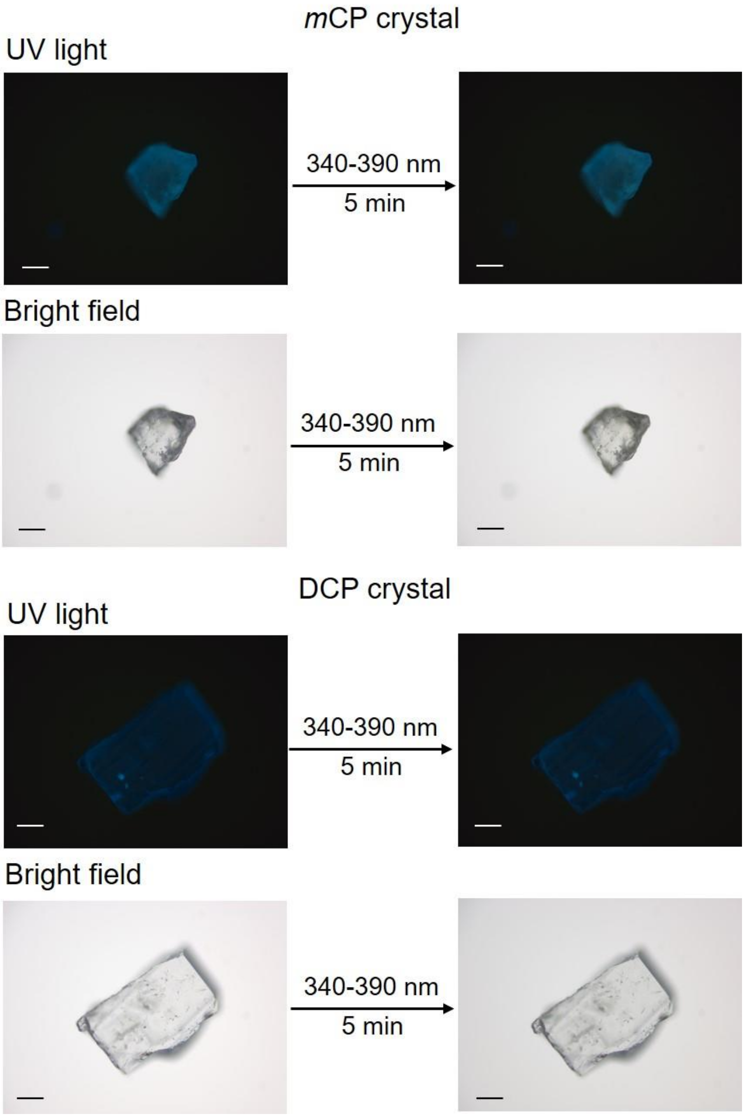

Fig. S1 Photographs of *m*CP and DCP crystal from the fluorescence microscope under UV irradiation, before (left) and after (right) irradiation (5 min), scale bar is 50 μm.

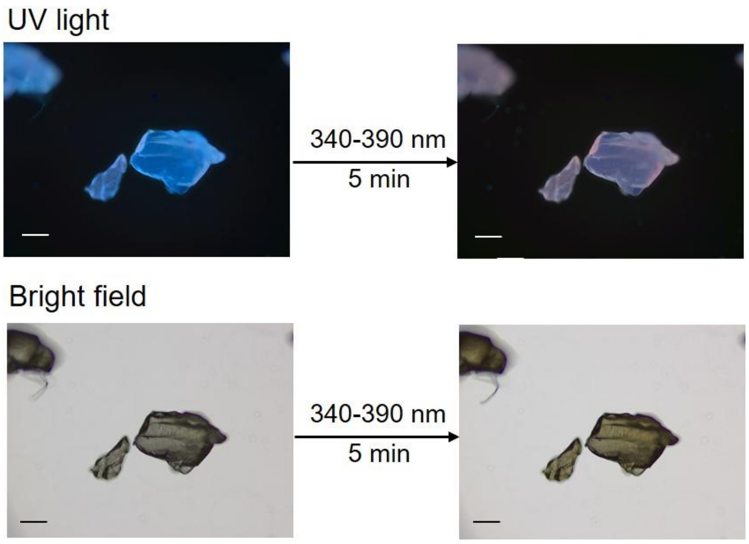

Fig. S2 Photographs DCP-DMA crystal from the fluorescence microscope under UV irradiation, before (left) and after (right) irradiation (5 min), scale bar is 50 μm.

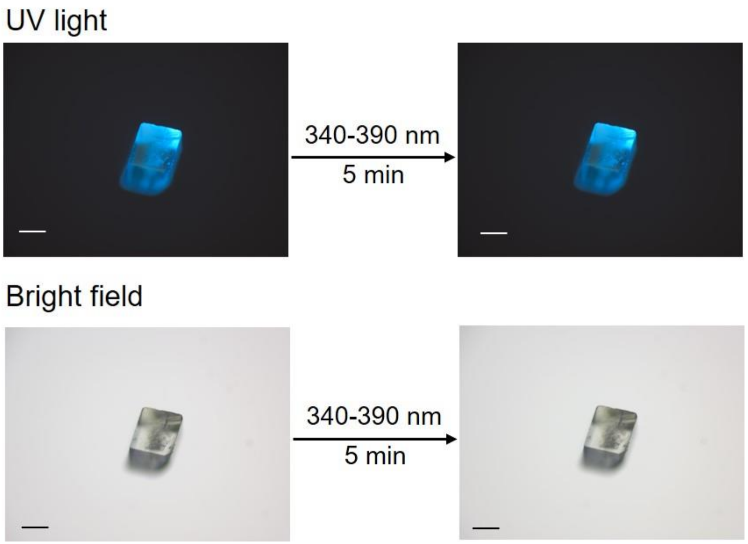

Fig. S3 Photographs TCP-DMA crystal from the fluorescence microscope under UV irradiation, before (left) and after (right) irradiation (5 min), scale bar is 50 μm.

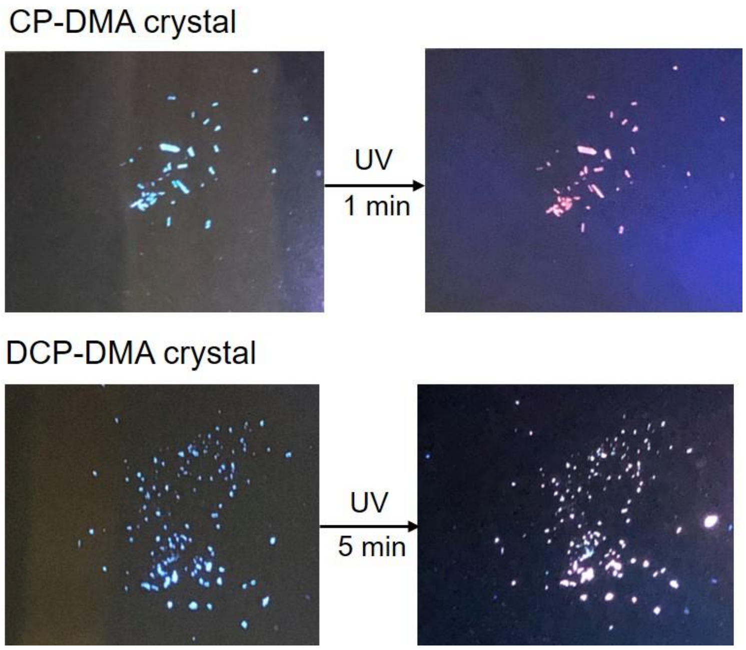

Fig. S4 Photographs of CP-DMA and DCP-DMA crystals taken under UV lamp irradiation (365 nm, 150 mW•cm^{-2}) before (left) and after (right) irradiation in the glove box.

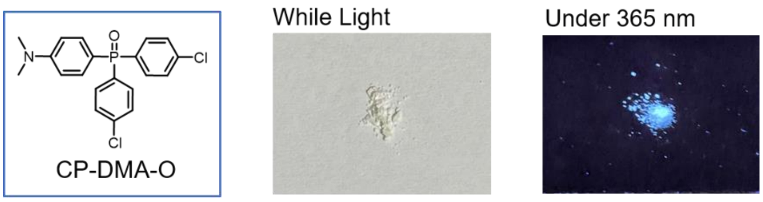

Fig. S5 Photographs of CP-DMA-O taken under while light and UV lamp (365 nm, 150 mW•cm^{-2}).

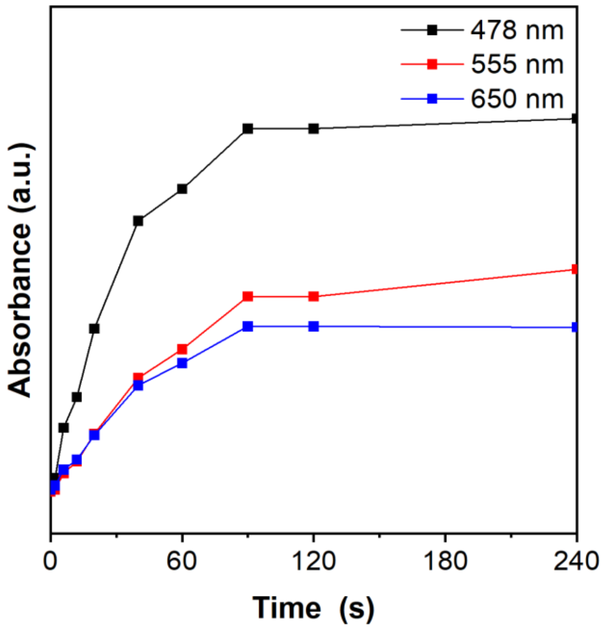

Fig. S6 Changes of absorbance of CP-DMA crystals versus time after UV irradiation at 478 nm, 555 nm and 650 nm (processed from Fig. 2b).

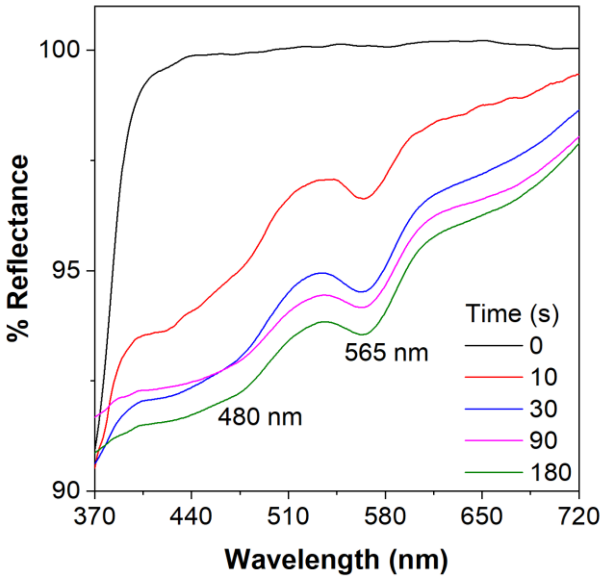

Fig. S7 Changes in the UV-Vis reflectance spectra of the DCP-DMA crystals upon irradiation at 365 nm (150 mW•cm^{-2}).

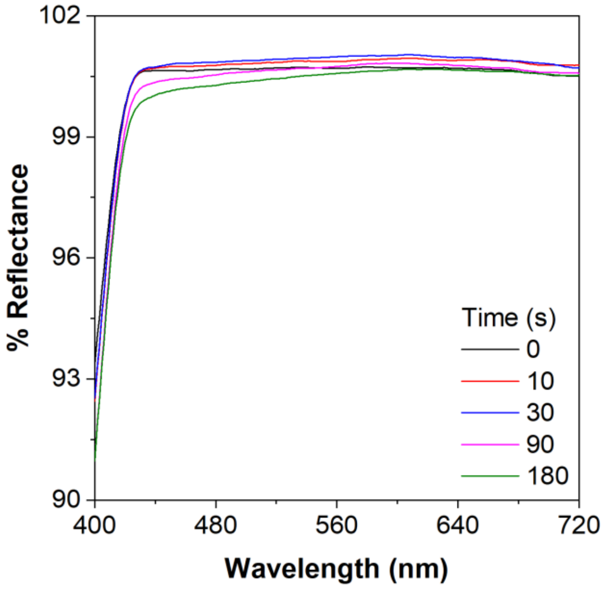

Fig. S8 Changes in the UV-Vis reflectance spectra of the TCP-DMA crystals upon irradiation at 365 nm (150 mW•cm^{-2}).

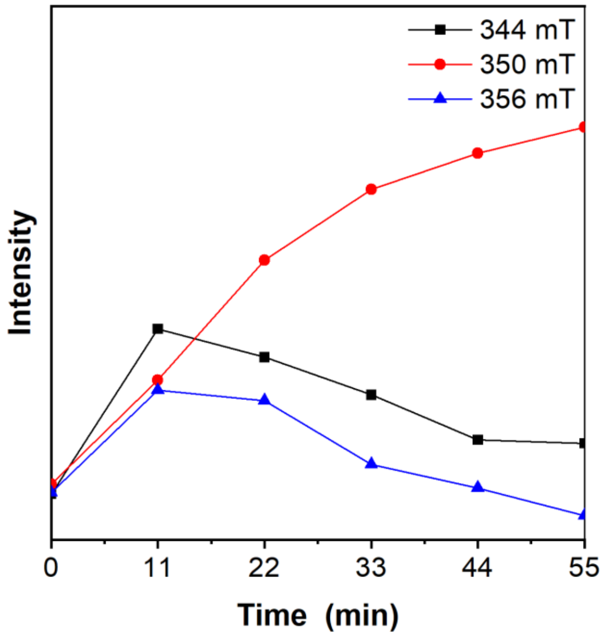

Fig. S9 Intensity changes of EPR signals at 344 mT, 350 mT and 356 mT of CP-DMA crystals after irradiation by UV lamp (365nm, 150 mW•cm^{-2}) for different time.

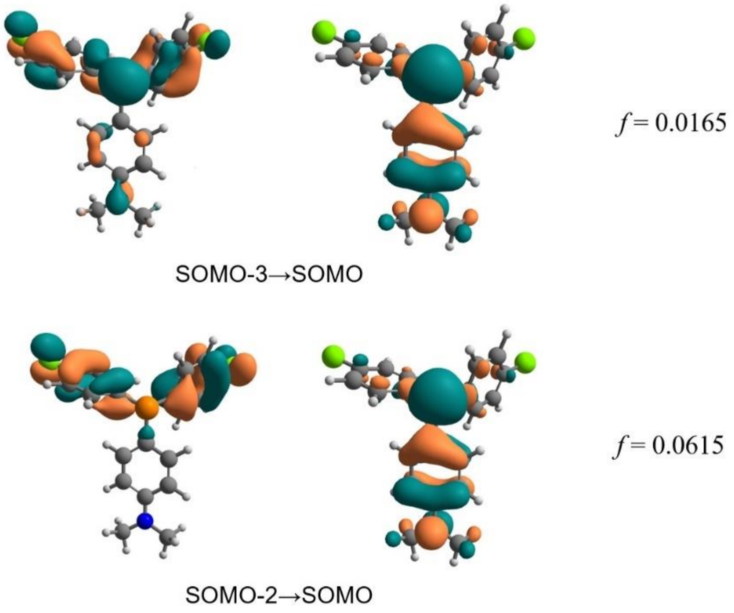

Fig. S10 [CP-DMA]$^{•+}$ orbitals relevant to optical transitions.

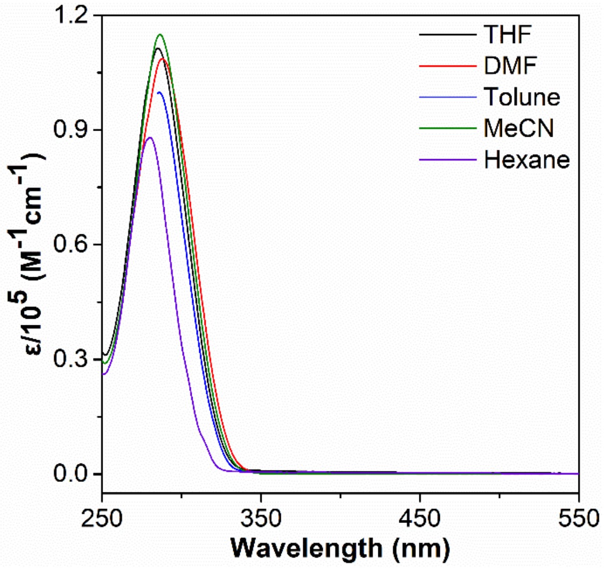

Fig. S11 Absorption spectra of CP-DMA in different solutions at 298 K (c = 5×10^{-5} M).

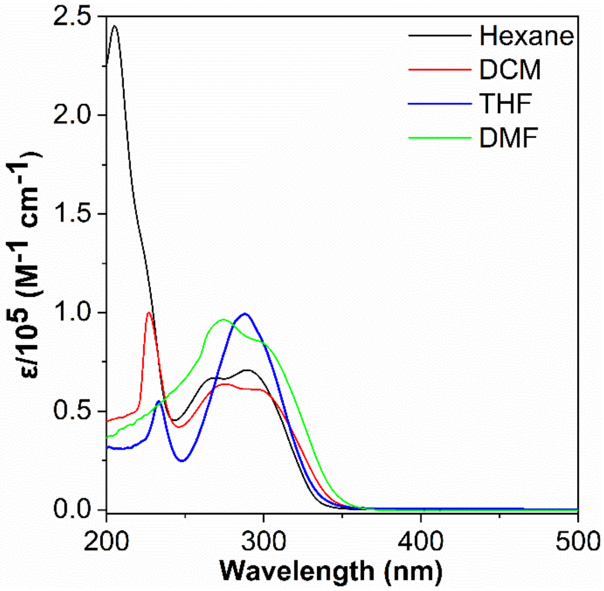

Fig. S12 Absorption spectra of DCP-DMA in different solutions at 298 K (c = 5×10^{-5} M).

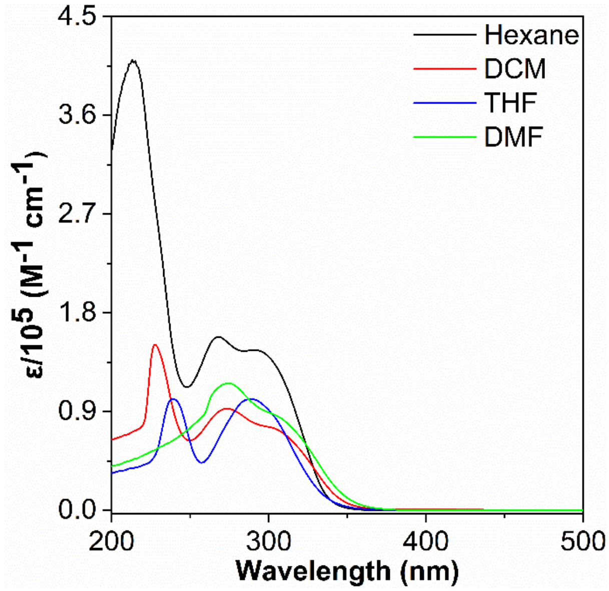

Fig. S13 Absorption spectra of TCP-DMA in different solutions at 298 K (c = 5×10^{-5} M).

Single crystal data

In the analysis of single crystals at the initial state and after UV irradiation, the single crystal data were collected at room temperature to avoid the possible influence of temperature, and the total collection time is about 40 min. Although molecular configurations and conformation changes upon UV irradiation were slight, the average data of the single crystal after UV irradiation was discernably different from the data recorded before UV irradiation. Thus, from this point, the molecular motions and the changes of molecular configurations and conformation could be double confirmed, as presented in the manuscript.

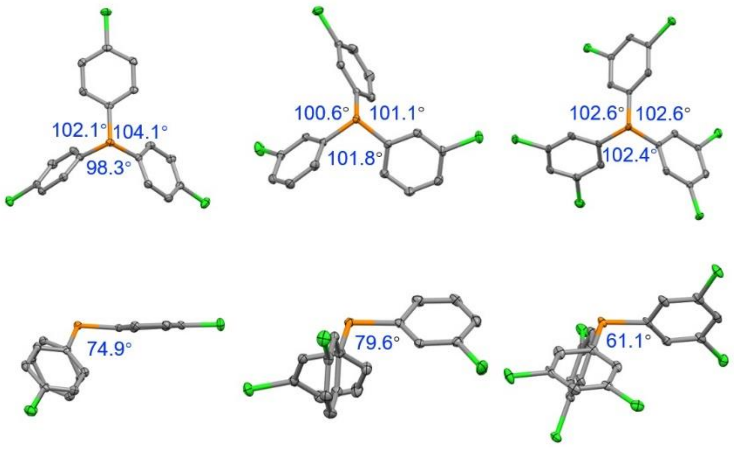

Fig. S14 Molecular conformations and selected angles of tris(4-chlorophenyl)phosphine, *m*CP, and DCP in the crystalline state with thermal ellipsoids at 50% probability. Hydrogen atoms are omitted for clarity. The dihedral angles of the average plane of the two phenyl rings and the last ring in tris(4-chlorophenyl)phosphine, *m*CP, and DCP single crystal.

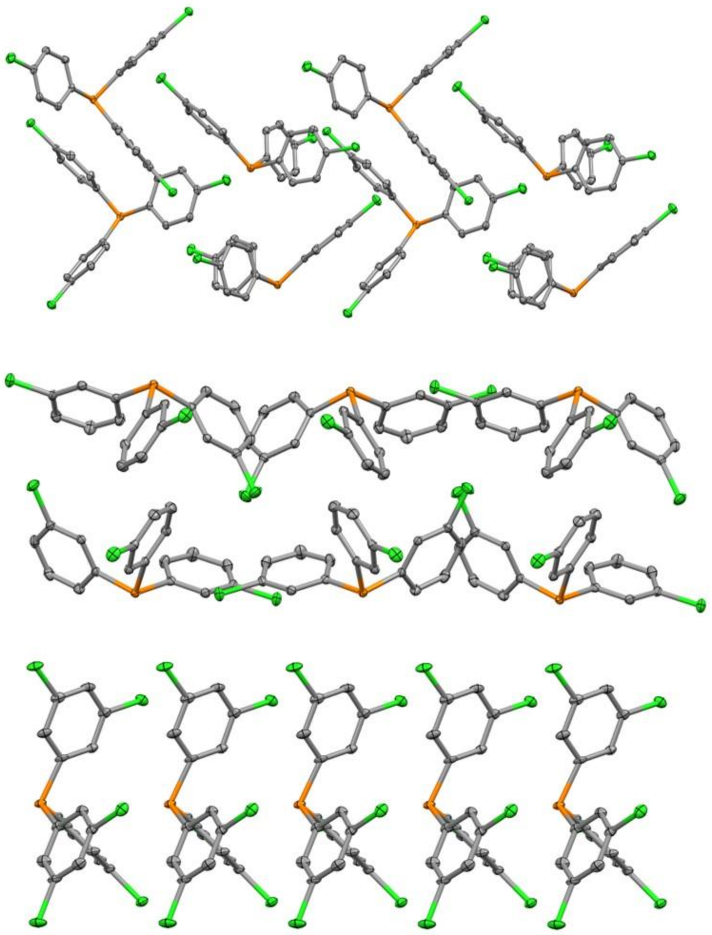

Fig. S15 Crystal packing diagrams of tris(4-chlorophenyl)phosphine (up), *m*CP (middle), and DCP (down).

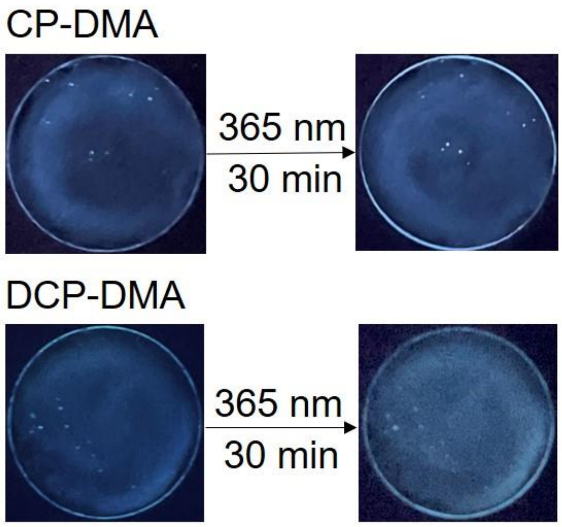

Fig. S16 Luminescence photographs of PVA films with doped CP-DMA and DCP-DMA (1 wt%) before and after the photoirradiation.

Table S1. Crystal data and structure refinement of *m*CP.

Empirical formula	C18 H12 Cl3 P
Formula weight	365.60
Temperature	150(2) K
Wavelength	1.54178 Å
Crystal system	orthorhombic
Space group	*P* bca
Unit cell dimensions	a = 15.8390(4) Å alpha = 90 deg.
	b = 10.3662(3) Å beta = 90 deg.
	c = 19.8011(5) Å gamma = 90 deg.
Volume	3251.15(15) Å^3
Z	8
Density (calculated)	1.494 Mg/m^3
Absorption coefficient	5.961 mm^{-1}
F(000)	1488
Crystal size	0.10 x 0.15 x 0.20 mm^3
Theta range for data collection	4.47 to 74.51
Index ranges	-16<=h<=19, -11<=k<=12, -24<=l<=23
Reflections collected	22665
Independent reflections	3304 [R(int) = 0.0402]
Absorption correction	Multi-scan
Max. and min. transmission	0.7580 and 0.3915
Refinement method	Full-matrix least-squares on F2
Data / restraints / parameters	3304 / 0 /199
Goodness-of-fit on F2	1.049
Final R indices [I>2sigma(I)]	R1 = 0.0297, wR2 = 0.0800
R indices (all data)	R1 = 0.0320, wR2 = 0.0783
Largest diff. peak and hole	0.420 and -0.553 e.Å^{-3}
CCDC reference	2214026

Table S2. Crystal data and structure refinement of DCP.

Empirical formula	C18 H9 Cl6 P
Formula weight	468.92
Temperature	293(2) K
Wavelength	1.54184 Å
Crystal system	triclinic
Space group	$P\bar{1}$
Unit cell dimensions	a = 4.8136(3) Å alpha = 98.197(2) deg.
	b = 12.6603(4) Å beta = 91.021 deg.
	c = 15.5459(3) Å gamma = 94.611(4) deg.
Volume	934.27(7) Å^3
Z	2
Density (calculated)	1.667 Mg/m^3
Absorption coefficient	9.193mm^{-1}
F(000)	468

Crystal size	0.02 x 0.01 x 0.01 mm^3
Theta range for data collection	2.873 to 76.594
Index ranges	-5<=h<=6, -15<=k<=15, -19<=l<=12
Reflections collected	9201
Independent reflections	3709 [R(int) = 0.0680]
Absorption correction	Multi-scan
Max. and min. transmission	0.6915 and 0.2367
Refinement method	Full-matrix least-squares on F2
Data / restraints / parameters	3709 / 0 /227
Goodness-of-fit on F2	1.087
Final R indices [I>2sigma(I)]	R1 = 0.0693, wR2 = 0.2418
R indices (all data)	R1 = 0.0798, wR2 = 0.2116
Largest diff. peak and hole	1.216 and -0.955 e.Å^{-3}
CCDC reference	2214027

Table S3. Crystal data and structure refinement of CP-DMA.

Empirical formula	C20 H18 Cl2 N P
Formula weight	374.25
Temperature	299(9) K
Wavelength	0.71073 Å
Crystal system	monoclinic
Space group	$P2_1/c$
Unit cell dimensions	a = 9.8297(5) Å alpha = 90 deg.
	b = 9.8673(4) Å beta = 99.078(4) deg.
	c = 20.3070(8) Å gamma = 90 deg.
Volume	1944.96(15) Å^3
Z	4
Density (calculated)	1.278 Mg/m^3
Absorption coefficient	0.417 mm^{-1}
F(000)	777.828
Crystal size	0.20 x 0.02 x 0.02 mm^3
Theta range for data collection	2.0370 to 28.4600
Index ranges	-12<=h<=11, -10<=k<=12, -25<=l<=28
Reflections collected	17021
Independent reflections	4898 [R(int) = 0.0266]
Absorption correction	Multi-scan
Max. and min. transmission	0.8634 and 0.5263
Refinement method	Full-matrix least-squares on F2
Data / restraints / parameters	4898 / 0 /220
Goodness-of-fit on F2	0.9977
Final R indices [I>2sigma(I)]	R1 = 0.0495, wR2 = 0.1609
R indices (all data)	R1 = 0.0744, wR2 = 0.1436
Largest diff. peak and hole	0.7466 and -0.6031 e.Å^{-3}
CCDC reference	2214023

Table S4. Crystal data and structure refinement of DCP-DMA.

Empirical formula	C20 H16 Cl4 N P
Formula weight	443.11
Temperature	293(2) K
Wavelength	0.71073 Å
Crystal system	triclinic
Space group	$P\bar{1}$
Unit cell dimensions	a = 9.5187(6) Å alpha = 91.822(4) deg.
	b = 10.5839(6) Å beta = 107.523(5) deg.
	c = 10.6683(5) Å gamma = 93.352(5) deg.
Volume	1021.77(10) $Å^3$
Z	2
Density (calculated)	1.442 Mg/m^3
Absorption coefficient	0.662 mm^{-1}
F(000)	452
Crystal size	0.11 x 0.02 x 0.02 mm^3
Theta range for data collection	2.456 to 76.522
Index ranges	-13<=h<=13, -13<=k<=15, -15<=l<=13
Reflections collected	15234
Independent reflections	5199 [R(int) = 0.0252]
Absorption correction	Multi-scan
Max. and min. transmission	0.7638 and 0.4063
Refinement method	Full-matrix least-squares on F2
Data / restraints / parameters	5199 / 0 /238
Goodness-of-fit on F2	1.088
Final R indices [I>2sigma(I)]	R1 = 0.0438, wR2 = 0.1297
R indices (all data)	R1 = 0.0586, wR2 = 0.1206
Largest diff. peak and hole	0.627 and -0.583 $e.Å^{-3}$
CCDC reference	2214024

Table S5. Crystal data and structure refinement of TCP-DMA.

Empirical formula	C20 H14 Cl6 N P
Formula weight	512.03
Temperature	236(4) K
Wavelength	0.71073 Å
Crystal system	triclinic
Space group	$P\bar{1}$
Unit cell dimensions	a = 9.8206(3) Å alpha = 84.150(2) deg.
	b = 10.8079(3) Å beta = 67.715(3) deg.
	c = 11.4388(3) Å gamma = 79.756(2) deg.
Volume	1104.73(6) $Å^3$
Z	2
Density (calculated)	1.5392 Mg/m^3
Absorption coefficient	0.858 mm^{-1}
F(000)	518.1103

Crystal size	0.10 x 0.05 x 0.02 mm^3
Theta range for data collection	1.9450 to 27.3400
Index ranges	-13<=h<=13, -14<=k<=14, -15<=l<=14
Reflections collected	17478
Independent reflections	5591 [R(int) = 0.0239]
Absorption correction	Multi-scan
Max. and min. transmission	0.7534 and 0.2063
Refinement method	Full-matrix least-squares on F2
Data / restraints / parameters	5591 / 0 /256
Goodness-of-fit on F2	0.9978
Final R indices [I>2sigma(I)]	R1 = 0.0461, wR2 = 0.1328
R indices (all data)	R1 = 0.0704, wR2 = 0.1194
Largest diff. peak and hole	0.4305 and -0.4367 e.$Å^{-3}$
CCDC reference	2214025

Table S6. Comparision of CP-DMA, DCP-DMA, TCP-DMA, and their irradiated *i*-CP-DMA, *i*-DCP-DMA, *i*-TCP-DMA single crystal parameters.

	CP-DMA	*i*-CP-DMA	DCP-DMA	*i*-DCP-DMA	TCP-DMA	*i*-TCP-DMA
d(P-C) (Å)[a]	1.817	1.813	1.814	1.813	1.815	1.815
θ_{AB} (deg)	85.52	85.73	87.56	87.57	89.34	89.63
θ_{BC} (deg)	89.38	89.46	71.85	71.84	86.43	86.38
	102.53	102.41	102.44	102.51	100.90	100.98
∠C-P-C(deg)	102.50	102.68	101.02	100.94	104.05	104.03
	100.84	100.72	101.45	101.31	102.62	102.67
∑(∠C-P-C) (deg)	305.87	305.81	304.91	304.76	307.57	307.68
Unit cell volume[b]	972.08	972.48	1021.77	1020.64	1104.73	1106.88

[a] d(P-C) refers to the bond length of phosphine and the pioneer carbon atom of *N,N*-dimethyl)aniline group.
[b] Cell formula units Z = 2.

EPR measurements.

EPR spectra were recorded with a Bruker ELEXSYS-II E500 spectrometer (X-band, MW frequency 9.8431~9.8480 GHz, MW power of 0.20 mW, modulation frequency of 100 kHz, and modulation amplitude of 0.50 mT) equipped with a high-Q cylindrical resonator ER4119HS.

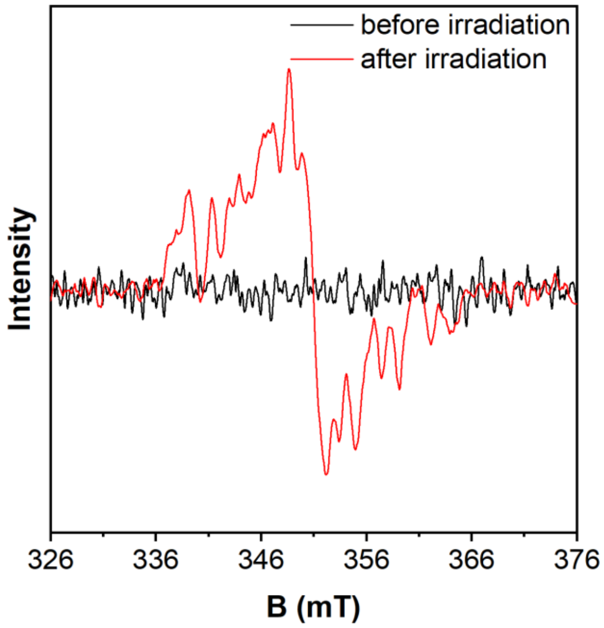

Fig. S17 EPR spectra of DCP-DMA crystals before and after irradiation (40 min).

Table S7. Parameters used in the simulations of the room-temperature EPR spectra of CP-DMA by "Spin Fit" function in Xepr software of Bruker ELEXSYS-II E500 Spectrometor.

$g_{\perp}$	2.00451
$A_{\perp}$ (G)	60.243
Linewidth$_{\perp}$ (G)	16.5954
$g_{\parallel}$	2.00547
$A_{\parallel}$ (G)	105.022
Linewidth$_{\parallel}$ (G)	24.4744

Table S8. Absolute spins calculation of D-A phosphines and tris(4-chlorophenyl)phosphine (*p*CP)[4]. The number of spins of molecules is calculated by the "Spin Count" function in Xepr software of Bruker ELEXSYS-II E500 Spectrometor.

Molecules	Q	MW frequency(GHz)	Mass (mg)	Spins/mg	Spins/mol
CP-DMA	11400	9.8469	3.2	4.9×10^{14}	3.04×10^{-4}
DCP-DMA	9100	9.8431	9.2	4.2×10^{14}	3.09×10^{-4}
TCP-DMA	9100	9.8468	6.8	none	-
*p*CP[4]	9800	9.8480	5.7	4.1×10^{14}	2.47×10^{-4}

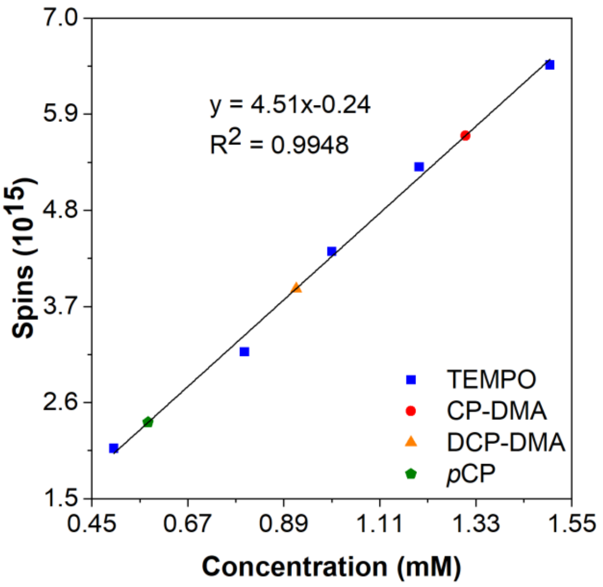

Fig. S18 Radical concentration determination. The TEMPO calibration curve is plotted, which covers the range of spin and concentration of CP-DMA, DCP-DMA, and *p*CP[4] in the photoinduced crystal samples (labeled with red, orange, and olive scatter, respectively).

Table S9. Absolute spins of standard solutions of TEMPO in degassed dry DCM and crystals of D-A phosphines and tris(4-chlorophenyl)phosphine (*p*CP[4]).

Molecules	Mass (mg)	Concentration (mM)	Spins ($\times 10^{15}$)
TEMPO	-	0.50	2.078
TEMPO	-	0.80	3.182
TEMPO	-	1.00	4.331
TEMPO	-	1.20	5.298
TEMPO	-	1.50	6.464
CP-DMA	3.5	-	5.655
DCP-DMA	6.8	-	3.901
*p*CP[4]	6.1	-	2.375

Computational details

All calculations were implemented in Gaussian 16[5]. Unrestrained geometry were optimized using the B3LYP functional including dispersion corrections GD3(BJ)[6], with the 6-31+G(d,p) basis set. TD-DFT calculations[7] were performed at the same B3LYP level of theory with the 6-311++G(d,p) basis set, including solvation energies evaluated by a self-consistent reaction field (SCRF) using an SMD implicit solvation model in toluene[8, 9]. The Gibbs energies were calculated at 298.15 K and 1 atm.

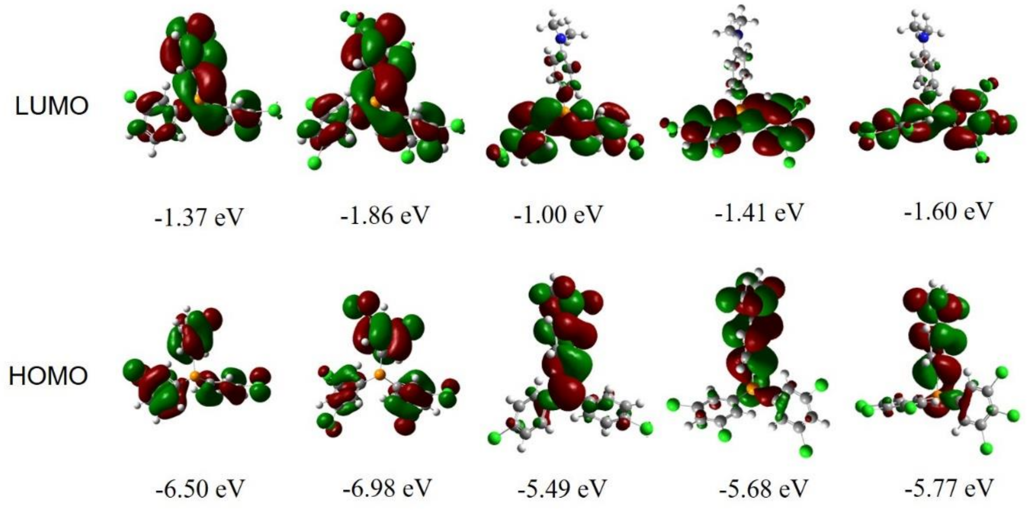

Fig. S19 Frontier molecular orbitals for *m*CP, DCP, CP-DMA, DCP-DMA and TCP-DMA.

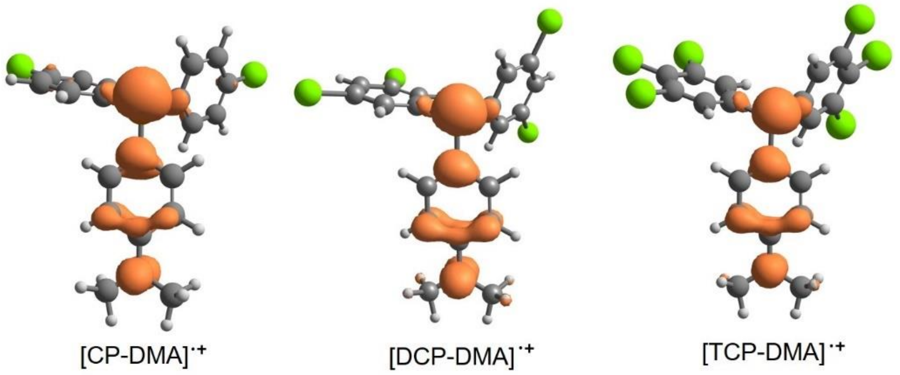

Fig. S20 Spin density distribution of CP-DMA, DCP-DMA and TCP-DMA radical cations in the gas state by DFT calculations (contour value = 0.003).

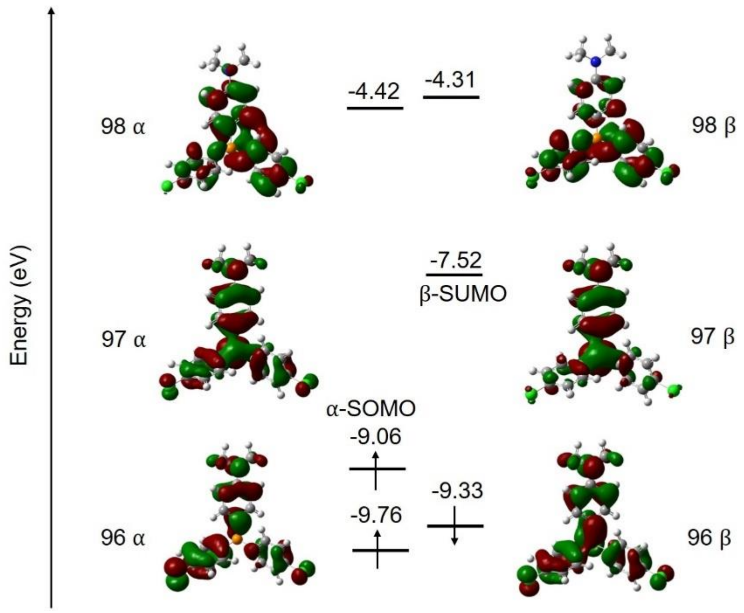

Fig. S21 FMOs of $[CP\text{-}DMA]^{\bullet+}$ from DFT calculations (isovalue=0.025).

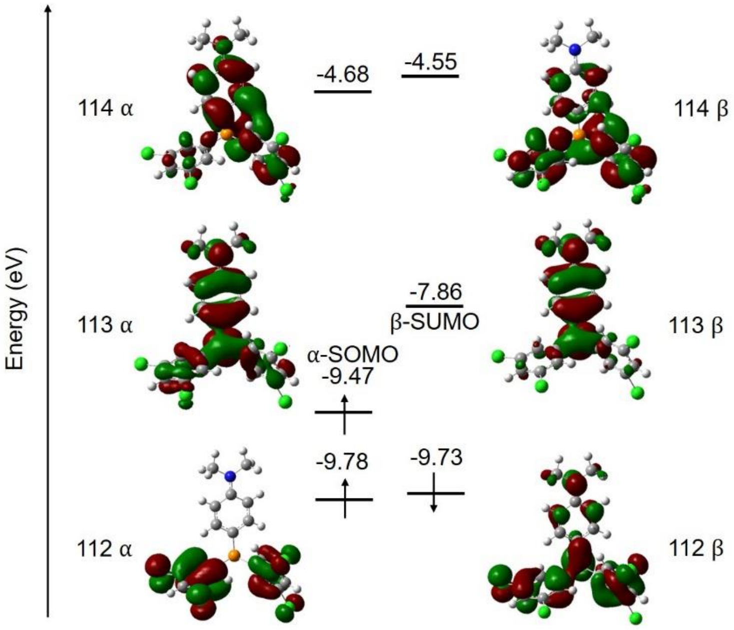

Fig. S22 FMOs of [DCP-DMA]$^{•+}$ from DFT calculations (isovalue=0.025).

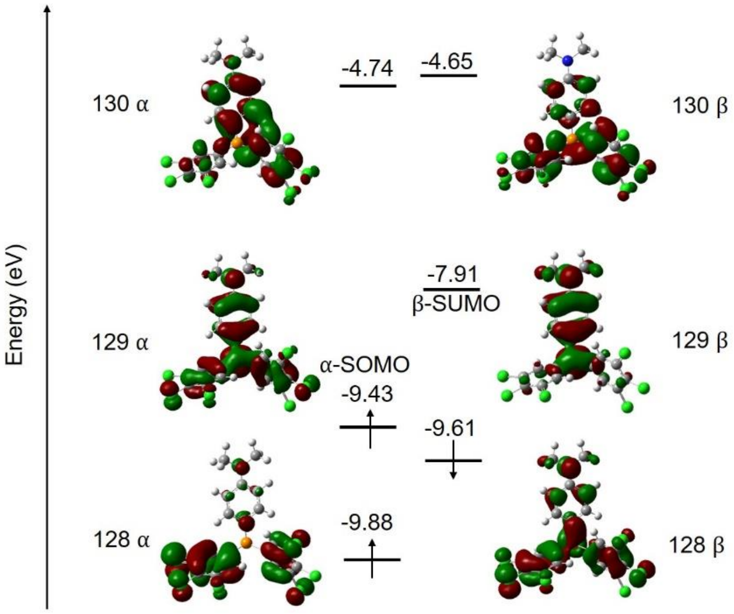

Fig. S23 FMOs of [TCP-DMA]$^{•+}$ from DFT calculations (isovalue=0.025).

Energies (Hartree) and Coordinates (Å)

[CP-DMA] $^{\cdot+}$

E(Done) = -2089.38277582

G= -2089.111022

P	-0.274001	0.000620	1.254813
Cl	-3.451257	5.055820	-0.866845
Cl	-3.182967	-5.227438	-0.832859
N	5.451379	0.110588	-0.465351
C	1.401447	0.025421	0.648651
C	-1.117415	-1.473365	0.629391
C	-1.128944	1.467706	0.629630
C	-0.837602	-2.715742	1.227434
H	-0.136359	-2.779988	2.054370
C	3.340775	-1.076093	-0.326313
H	3.760609	-1.945806	-0.813481
C	2.016560	-1.101992	0.049998
H	1.430607	-1.992342	-0.148325
C	-0.699824	2.162479	-0.515475
H	0.179588	1.834098	-1.058420
C	-1.462898	-3.873682	0.775030
H	-1.248351	-4.833392	1.230786
C	2.206760	1.169691	0.898377
H	1.772198	2.036881	1.385808
C	4.146759	0.082344	-0.100223
C	-2.559227	3.678260	-0.289591
C	-1.412726	3.265467	-0.976238
H	-1.092805	3.797705	-1.864813
C	3.529304	1.209976	0.525887
H	4.100799	2.106568	0.723849
C	-2.391109	-3.784922	-0.267330
C	-2.999667	3.001941	0.852405
H	-3.888185	3.338321	1.373996
C	-2.279058	1.903875	1.311845
H	-2.614931	1.384537	2.204345
C	-2.067741	-1.400432	-0.403467
H	-2.307950	-0.446895	-0.860754
C	-2.701194	-2.554534	-0.855617
H	-3.427876	-2.506083	-1.658530
C	6.081932	-1.061542	-1.078313
H	6.038699	-1.924628	-0.406430
H	7.126291	-0.835852	-1.281770
H	5.593117	-1.317990	-2.023854
C	6.266431	1.306129	-0.234231

H	5.836866	2.174271	-0.743764
H	7.265193	1.135753	-0.629818
H	6.349273	1.523051	0.835947

[CP-DMA] •⁻

E(Done) = -2089.62420825

G= -2089.359184

P	-0.269934	-0.002155	1.588244
Cl	-2.809792	5.312468	-0.987180
Cl	-3.752103	-4.726181	-0.974824
N	5.331237	-0.368528	-0.869632
C	1.394657	-0.089226	0.778808
C	-1.210374	-1.329211	0.799233
C	-0.953125	1.508524	0.758668
C	-1.509370	-2.494195	1.542083
H	-1.132330	-2.575612	2.559869
C	2.942356	-0.910620	-0.919061
H	3.099613	-1.442837	-1.852425
C	1.660095	-0.828448	-0.387564
H	0.842789	-1.336225	-0.889266
C	-0.147628	2.520691	0.223783
H	0.930156	2.395869	0.210334
C	-2.258975	-3.539814	1.020357
H	-2.461934	-4.431837	1.602602
C	2.485398	0.530943	1.413494
H	2.322429	1.087074	2.333930
C	4.037653	-0.278232	-0.280770
C	-2.093526	3.834286	-0.310428
C	-0.701010	3.699347	-0.298393
H	-0.068176	4.490341	-0.685360
C	3.786653	0.435739	0.896485
H	4.597370	0.916213	1.433767
C	-2.763358	-3.412412	-0.302211
C	-2.926834	2.834721	0.174666
H	-4.004615	2.950785	0.126386
C	-2.355381	1.671291	0.711528
H	-2.999898	0.886754	1.095180
C	-1.748002	-1.233174	-0.529590
H	-1.558348	-0.351617	-1.131127
C	-2.515565	-2.273037	-1.060244
H	-2.918957	-2.194474	-2.065971
C	5.817594	-1.723750	-1.111266

H	6.121140	-2.229045	-0.174164
H	6.684284	-1.686816	-1.780760
H	5.045770	-2.329871	-1.584648
C	6.365327	0.474472	-0.301854
H	6.021286	1.510970	-0.257458
H	7.249550	0.431594	-0.946193
H	6.672924	0.166518	0.716782

$[DCP\text{-}DMA]^{\bullet+}$
E(Done) = -3008.567972
G= -3008.3205

P	-0.087867	0.111366	-1.346784
N	5.560298	-1.058944	0.244992
C	1.574503	-0.228345	-0.777025
C	-0.644601	1.632237	-0.506733
C	-1.127461	-1.253752	-0.731607
C	-0.265020	2.862336	-1.064715
H	0.316571	2.909518	-1.978973
C	3.701018	0.494678	0.161121
H	4.282135	1.267777	0.645452
C	2.396768	0.763323	-0.184602
H	1.986030	1.742804	0.032658
C	-0.799847	-1.966732	0.432431
H	0.096582	-1.742326	0.996987
C	-0.659799	4.041783	-0.431898
C	2.154132	-1.496853	-1.057003
H	1.558020	-2.265507	-1.538314
C	4.277370	-0.789940	-0.092591
C	-2.839809	-3.272720	0.186012
C	-1.663003	-2.969319	0.872885
C	3.454049	-1.780661	-0.716511
H	3.849612	-2.763390	-0.934244
C	-1.442633	4.018053	0.723244
C	-3.146491	-2.550091	-0.968757
C	-2.299857	-1.547008	-1.442770
C	-1.438209	1.582732	0.647109
H	-1.749728	0.639076	1.077274
C	-1.825325	2.781183	1.247045
C	6.410352	-0.027047	0.849489
H	6.498300	0.839109	0.187049
H	7.403256	-0.438704	1.013972
H	6.002900	0.294789	1.813054
C	6.139128	-2.384526	-0.000597
H	5.555250	-3.159765	0.503921

H	7.153012	-2.406011	0.391840
H	6.175110	-2.600100	-1.073307
H	-3.501975	-4.052723	0.541577
H	-1.754179	4.939849	1.199053
Cl	-0.179323	5.577055	-1.106111
Cl	-2.806219	2.735541	2.688282
Cl	-1.266580	-3.859500	2.321340
H	-2.555686	-1.008294	-2.348335
Cl	-4.609694	-2.918389	-1.839394

[DCP-DMA]$^{\bullet-}$

E(Done) = -3008.838114
G= -3008.595535

P	-0.142628	0.000321	-1.616802
N	5.764035	0.001881	-0.003523
C	1.620028	0.000866	-1.037008
C	-0.805510	1.441924	-0.700900
C	-0.804363	-1.441923	-0.701085
C	-1.805256	2.221271	-1.336261
H	-2.019958	2.065230	-2.388924
C	3.694333	1.204608	-0.561201
H	4.187346	2.161899	-0.444654
C	2.337256	1.194713	-0.879283
H	1.820707	2.143387	-0.996336
C	-0.539776	-1.690052	0.679168
H	0.248964	-1.157655	1.195322
C	-2.503801	3.173827	-0.620287
C	2.337764	-1.192636	-0.879311
H	1.821667	-2.141553	-0.996448
C	4.407729	0.001470	-0.378343
C	-2.279828	-3.416874	0.750082
C	-1.276981	-2.647757	1.352390
C	3.694881	-1.201971	-0.561230
H	4.188291	-2.159060	-0.444774
C	-2.282934	3.415150	0.750735
C	-2.501221	-3.175253	-0.620841
C	-1.803607	-2.221862	-1.336578
C	-0.541328	1.689870	0.679539
H	0.247784	1.157960	1.195624

C	-1.279469	2.646665	1.352945
C	6.516973	1.228846	-0.195525
H	6.549376	1.555136	-1.250400
H	7.541107	1.074596	0.152015
H	6.089262	2.042518	0.396911
C	6.516564	-1.225937	-0.191240
H	6.087704	-2.037630	0.403082
H	7.540397	-1.071195	0.156968
H	6.550012	-1.555357	-1.245132
H	-2.858550	-4.141668	1.305495
H	-2.862329	4.139232	1.306362
Cl	-3.707240	4.184173	-1.455185
Cl	-0.929199	2.950251	3.071309
Cl	-0.926093	-2.951579	3.070593
H	-2.018589	-2.065561	-2.389143
Cl	-3.703983	-4.186252	-1.455768

[TCP-DMA] $^{\bullet+}$
E(Done) = -3927.744327
G= -3927.51982

P	-0.141662	0.310076	-1.476873
Cl	5.607204	-1.397388	0.628774
Cl	-4.329365	-3.726300	1.089281
N	-1.381850	5.852854	0.409630
C	-0.513680	1.934920	-0.821993
C	-1.310653	-0.860682	-0.714394
C	1.515806	-0.117829	-0.857923
C	-2.588010	-0.979384	-1.277797
H	-2.868941	-0.408446	-2.156164
C	-2.034816	3.526044	0.216276
H	-2.972174	3.712631	0.722757
C	-1.747759	2.245485	-0.197840
H	-2.469011	1.458140	-0.010358
C	2.033279	0.424068	0.326567
H	1.466965	1.137206	0.912601
C	-3.519399	-1.850918	-0.715582
C	0.400383	2.997843	-1.061184
H	1.339409	2.795711	-1.566628
C	-1.103987	4.591410	0.004349
C	4.054482	-0.912021	0.063368
C	3.290628	0.030561	0.779134
C	0.129565	4.280647	-0.651446
H	0.859954	5.055631	-0.840065

C	-3.182772	-2.639491	0.401641
C	3.520916	-1.448092	-1.125476
C	2.267175	-1.048891	-1.586029
H	1.889793	-1.471942	-2.510372
C	-0.955782	-1.646255	0.387936
H	0.031143	-1.582472	0.829069
C	-1.885322	-2.525956	0.939916
C	-2.664537	6.171959	1.046477
H	-3.498267	5.930436	0.380578
H	-2.694917	7.236426	1.266383
H	-2.779389	5.619410	1.984350
C	-0.414519	6.936921	0.206245
H	0.538827	6.698337	0.686540
H	-0.806213	7.848581	0.651080
H	-0.246983	7.113104	-0.861202
Cl	3.889020	0.723739	2.256632
Cl	4.413192	-2.610688	-2.053755
Cl	-5.097563	-1.952198	-1.433806
Cl	-1.408270	-3.473983	2.314264

[TCP-DMA]$^{\bullet -}$

E(Done) = -3928.023436
G= -3927.804567

P	0.101939	0.387214	-1.720779
Cl	2.110590	-5.274835	0.558292
Cl	-5.961187	0.396108	0.937338
N	4.158012	4.255217	0.670664
C	1.280589	1.581344	-0.956320
C	-1.447655	0.647392	-0.788224
C	0.695045	-1.206201	-0.974736
C	-2.460790	1.399699	-1.409153
H	-2.319601	1.786383	-2.414596
C	1.987651	3.127888	0.790132
H	1.755930	3.612640	1.729823
C	1.058124	2.236306	0.262799
H	0.136443	2.057497	0.806375
C	1.923154	-1.332891	-0.318534
H	2.551131	-0.466381	-0.151860
C	-3.638882	1.663016	-0.714867
C	2.468447	1.879253	-1.639432
H	2.660525	1.414747	-2.603951
C	3.203145	3.398027	0.121873
C	1.570488	-3.725628	-0.022633

C	2.352252	-2.575462	0.146190
C	3.414885	2.760023	-1.120907
H	4.312792	2.951761	-1.694525
C	-3.881939	1.191816	0.568684
C	0.333144	-3.587897	-0.677160
C	-0.094557	-2.352558	-1.149728
H	-1.060668	-2.284079	-1.638066
C	-1.650922	0.190241	0.527930
H	-0.877699	-0.366042	1.047991
C	-2.853227	0.473286	1.167612
C	3.763987	5.118005	1.772503
H	2.952533	5.813742	1.502099
H	4.629484	5.701227	2.092339
H	3.432027	4.525173	2.629795
C	5.228743	4.738242	-0.183514
H	5.825092	3.904808	-0.567553
H	5.893632	5.372694	0.405471
H	4.860633	5.321488	-1.044547
Cl	3.903120	-2.664031	0.961395
Cl	-0.701675	-4.978730	-0.918541
Cl	-4.852331	2.685826	-1.534452
Cl	-3.021902	-0.086914	2.854978

References

1 S. Postle, V. Podgorny and D. W. Stephan, *Dalton Transactions*, 2016, **45**, 14651-14657.

2 S. Xie, Z.-J. He, L.-H. Zhang, B.-L. Huang, X.-W. Chen, Z.-S. Zhan and F.-M. Zhang, *Chemical Communications*, 2021, **57**, 2069-2072.

3 M. Narsireddy and Y. Yamamoto, *The Journal of Organic Chemistry*, 2008, **73**, 9698-9709.

4 X. Zhao, J. Gong, P. Alam, C. Ma, Y. Wang, J. Guo, Z. Zeng, Z. He, H. H. Y. Sung, I. D. Williams, K. S. Wong, S. Chen, J. W. Y. Lam, Z. Zhao and B. Z. Tang, *CCS Chem.*, 2022, **4**, 1912-1920.

5 M. J. Frisch, H. B. Schlegel, G. E. Scuseria, M. A. Robb, J. R. Cheeseman, G. Scalmani, V. Barone, B. Mennucci, G. A. Petersson, H. Nakatsuji, M. Caricato, X. Li, H. P. Hratchian, A. F. Izmaylov, J. Bloino, G. Zheng, J. L. Sonnenberg, M. Hada, M. Ehara, K. Toyota, R. Fukuda, J. Hasegawa, M. Ishida, T. Nakajima, Y. Honda, O. Kitao, H. Nakai, T. Vreven, J. A. Montgomery Jr., J. E. Peralta, F. Ogliaro, M. Bearpark, J. J. Heyd, E. Brothers, K. N. Kudin, V. N. Staroverov, R. Kobayashi, J. Normand, K. Raghavachari, A. Rendell, J. C. Burant, S. S. Iyengar, J. Tomasi, M. Cossi, N. Rega, J. M. Millam, M. Klene, J. E. Knox, J. B. Cross, V. Bakken, C. Adamo, J. Jaramillo, R. Gomperts, R. E. Stratmann, O. Yazyev, A. J. Austin, R. Cammi, C. Pomelli, J. W. Ochterski, R. L. Martin, K. Morokuma, V. G. Zakrzewski, G. A. Voth, P. Salvador, J. J. Dannenberg, S. Dapprich, A. D. Daniels, Ö. Farkas, J. B. Foresman, J. V. Ortiz, J. Cioslowski, D. J. Fox, *Gaussian 16*, Gaussian, Inc., Wallingford CT, 2016.

6 S. Grimme, J. Antony, S. Ehrlich and H. Krieg, *J. Chem. Phys.*, 2010, **132**, 154104.

7 C. Adamo and D. Jacquemin, *Chem. Soc. Rev.*, 2013, **42**, 845-856.

8 A. V. Marenich, C. J. Cramer and D. G. Truhlar, *J. Phys. Chem. B*, 2009, **113**, 4538-4543.

9 A. V. Marenich, C. J. Cramer and D. G. Truhlar, *J. Phys. Chem. B*, 2009, **113**, 6378-6396.
